# Detection of Potential Problematic *Cytb* Gene Sequences of Fishes in GenBank

**DOI:** 10.3389/fgene.2018.00030

**Published:** 2018-02-06

**Authors:** Xiaobing Li, Xuejuan Shen, Xiao Chen, Dan Xiang, Robert W. Murphy, Yongyi Shen

**Affiliations:** ^1^College of Veterinary Medicine, South China Agricultural University, Guangzhou, China; ^2^College of Marine Sciences, South China Agricultural University, Guangzhou, China; ^3^Joint Influenza Research Centre (SUMC/HKU), Shantou University Medical College, Shantou, China; ^4^Centre for Biodiversity and Conservation Biology, Royal Ontario Museum, Toronto, ON, Canada; ^5^Key Laboratory of Zoonosis Prevention and Control of Guangdong Province, Guangzhou, China

**Keywords:** sequence error, DNA barcoding, fish, GenBank, *Cytb*

## Abstract

Fishes are, by far, the most diverse group of vertebrates. Their classification relies heavily on morphology. In practice, the correct morphological identification of species often depends on personal experience because many species vary in their body shape, color and other external characters. Thus, the identification of a species may be prone to errors. Due to the rapid development of molecular biology, the number of sequences of fishes deposited in GenBank has grown explosively. These published data likely contain errors owing to invalid or incorrectly identified species. The erroneous data can lead to downstream problems. Thus, it is critical that such errors get identified and corrected. A strategy based on DNA barcoding can detect potentially erroneous data, especially when intraspecific K2P variation exceeds interspecific K2P divergence. Analyses of the most used DNA marker for fishes (mitochondrial *Cytb*) discovers that intraspecific differences of fishes are generally less than 1%, while interspecific differences are generally higher than 10%. Based on this ruler, our analyses identify 1,303 potential problematic *Cytb* sequences of fishes in GenBank and point to taxonomic problems, errors in identification, genetic introgression and other concerns. Care must be taken to avoid the perpetuation of errors when using these available data.

## Introduction

The identification of fishes generally relies on morphology and distribution. However, in practice, problems exist due to the great diversity of fishes, small body sizes of many species, poor preservation of individual specimens and other issues. Further, accuracy in the morphological identification of species depends on personal experience. For many species, abiotic factors such as environmental pertubations can affect body shape, skin color and other external characters (Wilkens and Strecker, 2003). These factors inevitably lead to controversy and misidentification.

DNA barcoding uses a short gene segment to identify species (Hebert et al., 2003a, 2004). Generally, mitochondrial *COI* gene is the marker of choice because differences in sequences between species have been well characterized (Hebert et al., 2003b). This method has been applied to the classification of fishes to facilitate the rapid and accurate identification of species and the discovery of the cryptic species (Fields et al., 2015; Bhattacharya et al., 2016). In DNA barcoding, a short standardized sequence can distinguish individuals of a species because genetic variation between species usually exceeds that within species (Hebert et al., 2003a; Hajibabaei et al., 2007). In such cases, any gene segment can serve to identify species. Potential errors and taxonomic conundrums can be identified when interspecific genetic variation does not exceed that within species.

Because of advances in sequencing technologies, the number of DNA sequences of fishes has increased explosively in GenBank. For example, fishes now have more than 60,000 sequences of mitochondrial cytochrome *b* (*Cytb*) alone in the database, and this representation is ever increasing. Many sequences have been submitted by labs void of taxonomic expertise. Further, sampling error, contamination, hybridization, introgression, and nuclear pseudogenes can also lead to problems and errors. Consequently, any large database likely contains errors and the perpetuation of erroneous data can lead to downstream problems. Thus, it is critical to identify and correct such errors.

The large gap between *Cytb* intra- and interspecies differences is stable. Consequently, the gene has been used widely in systematics and molecular ecology including the identifications of species of chickens, praomyin rodents and gadid fishes, among many others (Kartavtsev, 2011; Nicolas et al., 2012; Yacoub et al., 2015; Fernandes et al., 2017). Many studies on fishes have used *Cytb* sequences for molecular phylogenetics and population analyses. Therefore, we use *Cytb* to test if DNA barcoding can identify potential erroneous sequences of fishes. This approach has the potential to be used universally to improve the quality of publically available data.

## Materials and methods

To obtain the maximum number of sequences, we downloaded all 65,326 *Cytb* records for fishes from NCBI. These sequences, which were uploaded by many labs, many of them were incomplete *Cytb* genes, had different lengths and covered different parts of the gene. Therefore, we employed the following trimming steps to standardize these sequences before calculating sequence divergences: (1) flanking regions of *Cytb* were deleted; (2) sequences were aligned using MAFFT (Katoh and Toh, 2010); (3) to obtain the maximum number of homologous sequences, we balanced the maximum length alignment vs. taxonomic coverage to attain the final trimmed dataset for downstream analyses. The trimmed dataset consisted of 35,130 fragments of 918 bp. When we set the complete *Cytb* for *Carassius auratus* GU135519.1 as the standard, the available fragments ranged from 75 to 998 bp.

DAMBE (Xia and Xie, 2001) was employed to detect for nucleotide substitution saturation. Iss < ss.c was statistically significant (*P* = 0), indicating that the nucleotide substitution was not saturated (Xia et al., 2003). Pairwise divergences (Kimura 2-parameter, K2P) of these sequences were calculated using MEGA 6 (Tamura et al., 2013). Then, intraspecific distances greater than 1% and interspecific distances less than 10% were identified as being potentially problematic. Neighbor-joining trees with 1,000 bootstrap replications were constructed using MEGA 6 (Tamura et al., 2013) to visualize similarity and sequence divergence. Sequences with intraspecific K2P divergences greater than interspecific differences were retained for further evaluation.

## Results and discussion

The compiled a dataset of *Cytb* sequences of fishes from GenBank exhibited a great diversity of lengths. A clear tradeoff existed between maximizing the length of the alignments and taxonomic coverage (Shen et al., 2013). Usable fragment lengths ranged from 55 to 972 bp. Our final dataset consisted of 35,130 fragments of 918 bp. We regarded GenBank accession number GU135519.1 for *Cytb* to be the standard for all comparisons.

**Figure 1:**
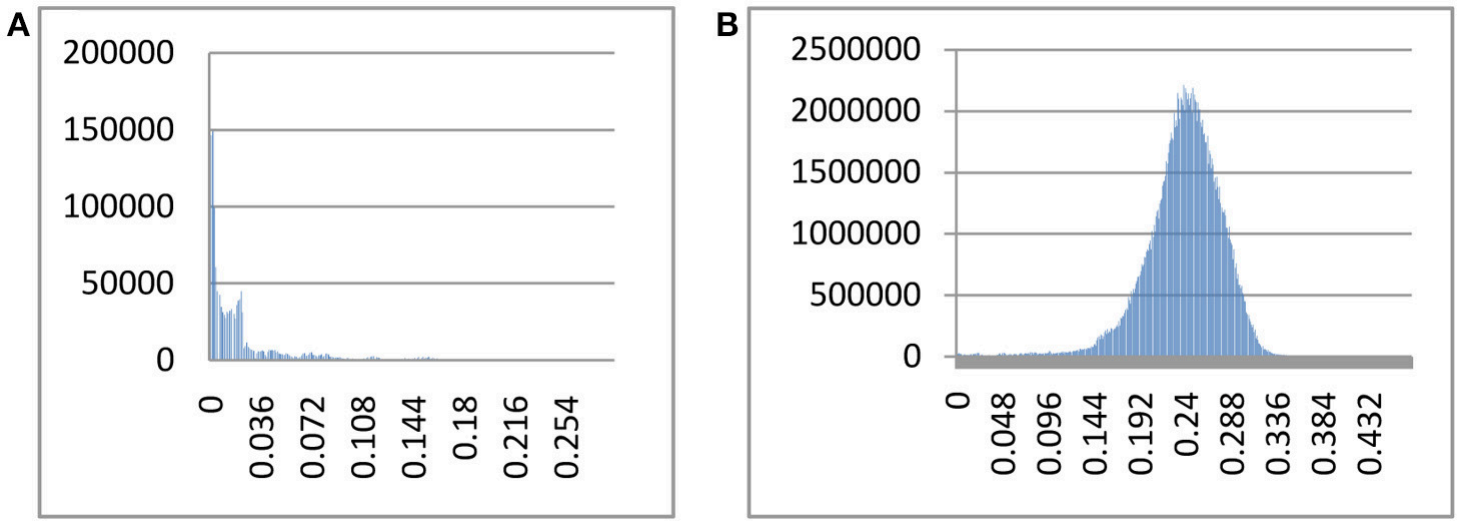
Intra- and interspecific pairwise divergence (Kimura 2-parameter) of *Cytb* in fishes. **(A)** Intraspecific divergence. **(B)** Interspecific divergence.

The index of substitution saturation (Iss) is significantly less than the critical Iss.c (*P* = 0) (Table 1). This result suggests that the nucleotide substitutions are not saturated. The distribution of genetic distances was shown to vary greatly (Johns and Avise, 1998). Notwithstanding, our intraspecific differences generally fall below 1%, while interspecific differences usually exceed 10% (Figure 1). The gap suggests that *Cytb* can efficiently distinguish different species of fishes. Some notable exceptions exist. For example, sequences with shallow interspecific divergence (<10%), deep intraspecific divergence (>1%), and interspecific differences that are much less than intraspecific differences constitute potential errors. Based on this ruler, we identify 1,303 potential problematic *Cytb* gene sequences (Table S1).

**Table 1:** Test of substitution saturation of *Cytb* sequences of fishes.

	**Iss**	**I ss.cSym**	**T**	**DF**	**P**	**Iss.cSym**	**T**	**DF**	**P**
4	0.298	0.817	29.382	917	0.000	0.785	27.583	917	0.000
8	0.296	0.784	24.705	917	0.000	0.677	19.302	917	0.000
16	0.294	0.766	22.614	917	0.000	0.565	12.988	917	0.000
32	0.299	0.742	20.696	917	0.000	0.431	6.190	917	0.000

Shallow interspecific divergence may owe to several possibilities. (1) Species of recent origin should have very shallow interspecific divergence. For example, the K2P divergence between *Comephorusdy bowskii* and *C. baicalensis* is only 0.4–1.0%, and between *Etheostoma kanawhae* and *E. osburni* a mere 0.4–0.7%. These species appear to have recent origins (Syu et al., 1994; Sun et al., 2007; Geiger et al., 2016). (2) MtDNA introgression can lead to shallow interspecific differences. For example, *Melanotaenia misoolensis* (KC133624.1) is very similar to *M. flavipinnis* (0.2–0.3%), and *M. boesemani* (KC133618.1) shows shallow interspecific divergence with *M. ajamaruensis* (0.3–0.4%). Gene introgression via hybridization occurs in rainbowfishes (Unmack et al., 2013). The low genetic divergence between *Chasmistes brevirostris* and *Deltistes luxatus*is (0.8–1%) is also due to introgressive hybridization (Dowling et al., 2016). Introgressive hybridizations were also found in suckers, darters, barbs and so on (Near et al., 2011; Unmack et al., 2014; Bernal et al., 2017; Schmidt et al., 2017). This reason leads to the unexpected shallow interspecific divergence in many fishes. Nuclear sequences would be helpful to classify recent origin or mtDNA introgression. (3) Errors in species identification and conspecificity of the species can also lead to low values of divergence. For example, *Etheostoma spectabile* (FJ381067.1, FJ381066.1, FJ381061.1, and FJ381057.1), *E. bison* (KF377137.1), *E. burri* (FJ381080.1 and AY374262.1), and *E. lawrencei* (KF377157.1 and KF377156.1) show shallow interspecific divergence with *E. caeruleum* (0.3–1.1%). This result suggests conspecificity of the species, or species misidentifications. K2P distances between *Etheostoma sitikuense, E. percnurum, E. marmorpinnum* range from 0.2 to 1.1%. The low levels of interspecific divergence indicate either recent divergence or perhaps a taxon-specific slowing of the molecular clock. Although no specific level of divergence can identify species, low interspecific divergence point to a need for further investigation.

Larger than expected intraspecific differences also exist. For example, two sequences of *Paramisgurnus dabryanu* (KM186183.1, KF771003.1) differ from conspecifics by 18.1–19.6%, one *Paracobitis malapterura* (LC167412.1) differs by 22.0–22.4%, two *Etheostoma coosae* (HQ128114.1, AY374266.1) by 10.9–12.2%, two *Rhodeus ocellatus* (KT004415.1, AF051876.1) by 20.0–20.6%, and two *Schizothorax waltoni* (KT833090.1, KT833089.1) by 19.2–20.7%. These cases indicate that at least half of the sequences were either incorrectly identified to species, contamination of DNA occurred in the laboratory, or an errneous sequence was submitted to GenBank.

Species having wide ranges of intraspecific differences are most likely composites of multiple cryptic species. For example, *Etheostoma nigripinne* has complex relationships, and its intraspecific divergences range from 0.0 to 14.5%. Similarly, intraspecific divergences of *E. rufilineatum* range from 0.1 to 12.6%. Many currently recognized species contain a few cryptic species (Köhler et al., 2005; Palandacic et al., 2017; Phuong et al., 2017). Further taxonomic study is necessary for those species with wide ranges of intraspecific differences.

**Figure 2:**
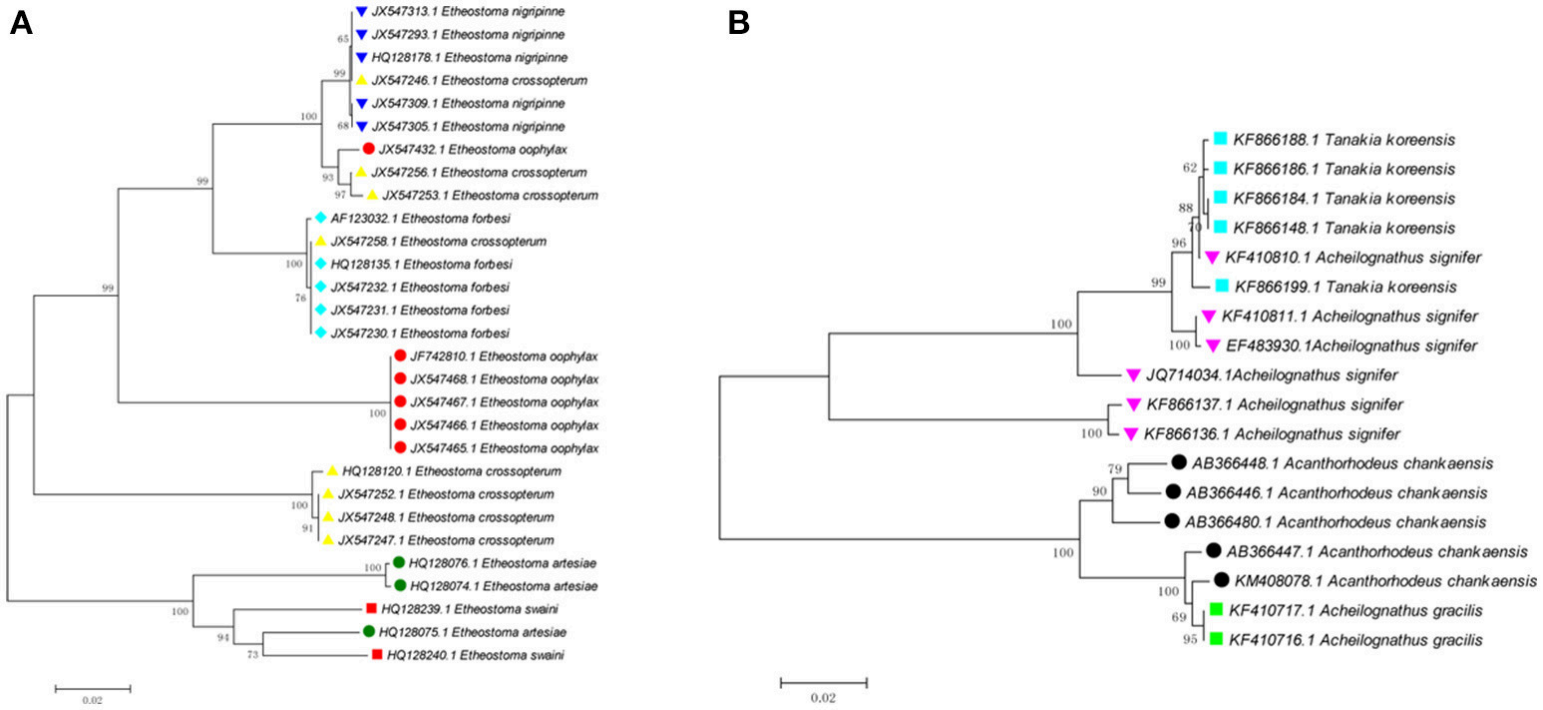
Two examples of potential errors for *Cytb* sequences in fishes.

Cases where interspecific differences are much less than intraspecific differences likely owe to problems such as species misidentifications, database errors when submitting sequences to GenBank, laboratory mix-ups, laboratory contamination, and other issues. For example, one sequence of *Etheostoma oophylaxe* (JX547432.1) has shallow interspecific divergence with *E. nigripinne* (0.1–4.1%), but deep intraspecific divergence (13.8–14.5%) (Figure 2A). One sequence of *E. artesiae* (HQ128075.1) has relatively low interspecific divergences with *E. swaini* (5.4–7.6%), but deep intraspecific divergence (10.3–10.4%). Three sequences of *E. crossopterum* (JX547246.1; JX547256.1; and JX547253.1) have shallow interspecific divergence with *E. nigripinne* (0–0.3%) but exhibit deep intraspecific divergence (15.6–16.8%). Four sequences of *Acheilognathus signifier* (KF410810.1; KF410811.1; EF483930.1 and JQ714034.1) have low interspecific divergences with *Tanakia koreensis* (0.2–5.2%), yet deep intraspecific divergence (15.4–16.1%; Figure 2B). Further investigation into the discordance is desirable.

Other reasons can lead to unexpected values of genetic divergence. (1) Great geographic distances can result in genetic divergence, especially in widely distributed species. (2) Recent origins of species can result in high levels of genetic similarity. (3) Taxonomic change can result in errors. For example, the names *Rutilus lemmingii* and *Chondrostoma lemmingii* differ, but they are the same species, as do *Epinephelus lanceolatus* and *Promicrops anceolatus*. Therefore, we suggest that GenBank (NCBI) provide a mechanism for updating changes in taxonomic classification. (4) Morphologically different species may have essentially identical genes. For example, many species of darters (*Etheostoma*) differ morphologically, but genetically differ slightly. Similarly, *Glossolepis incisus, G. pseudoincisus*, and *G. dorityi* are all essentially identical genetically (Unmack et al., 2013). It has to be mentioned that without standard sequences for each species, when two sequences have atypical genetic divergence values, we cannot classify which sequence is correct and which is wrong. Further investigations into species with atypical genetic divergence values (Table S1) can improve the accuracy of the fish mitochondrial database and foster interesting study.

DNA barcoding can complement morphological classifications and provide an alternative approach to assessing species diversity. Now, the approach is widely used to identify species of fishes (Ward et al., 2005; Smith et al., 2008; Ardura et al., 2010; Filonzi et al., 2010). Classifications form the basis of evolutionary research and incorrect taxonomies can negatively affect all other biological investigations. Fishes comprise nearly half of all vertebrate species, and, thus, an accurate classification is essential. Species identification errors in GenBank can mislead subsequent research. We detect potentially problematic data for one gene only, *Cytb*, for sequences from fishes. The approach will be useful for other mitochondrial genes and other taxa. DNA barcoding can identify species of fishes, species complexes, sister-species, and discover potentially problematic errors.

## Author contributions

XL carried out the data analysis and drafted the manuscript; XS, XC, and DX carried out data analysis; YS designed and coordinated the study, and helped draft the manuscript; RM revised the manuscript. All authors gave final approval for publication.

### Conflict of interest statement

The authors declare that the research was conducted in the absence of any commercial or financial relationships that could be construed as a potential conflict of interest.
