# Supplementary material for: Detection of Potential Problematic Cytb Gene Sequences of Fishes in GenBank
Source: Front Genet. 2018 Feb 6;9:30. doi: 10.3389/fgene.2018.00030 (PMC5808227; doi:10.3389/fgene.2018.00030)
Supplement: Supplementary file 1 [file Table1.docx]

Table S1. Potential **problematic** *Cytb* sequences in fishes

| Potential problematic sequences | reasons |
| --- | --- |
| KF955012.1*Abbottina obtusirostris* | Interspecific divergence with *Abbottina rivularis* (0.2%–0.3%) is much less than intraspecific divergence (20.0%–20.3%) |
| KU052314.1–KU052316.1; EU934493.1–EU934495.1; KP645250.1–KP645253.1; LC098134.1; KP645245.1; KP645246.1; EU934499.1; LC114486.1; AB794026.1; *Abbottina rivularis* | Deep intraspecific divergence (10.5%–15.9%) |
| AB366444; AB366443.1; AB366445.1 *Acheilognathus barbatulus* | Interspecific divergence with *Acheilognathus rhombeus* (0.7%–3.6%) is much less than intraspecific divergence (12.3%–13%) |
| KP015738.1 *Acheilognathus imberbis* | Deep intraspecific divergence (19.7%–20.2%) |
| EF571663.1; EF571662.1; EF571657.1; AB366453.1 *Acheilognathus macropterus* | Deep intraspecific divergence (11.3%–11.7%) |
| KF410698.1 *Acheilognathus asmussii* | Shallow interspecific divergence with *Acheilognathus macropteru* (0.1%–2.6%) |
| AB366483.1; AB366482.1 *Acheilognathus peihoensis* | Shallow interspecific divergence with *Acheilognathus barbatulus*  (0.5%–0.9%) |
| EU636235.1; EU636234.1 *Acheilognathus tonkinensis* | Interspecific divergence with *Acheilognathus rhombeus* (1.5%–3.6%) is much less than intraspecific divergence (13.4%–14%) |
| KF410810.1; KF410811.1; EF483930.1; JQ714034 *Acheilognathus signifer* | Interspecific divergence with *Tanakia koreensis* (0.2%–5.2%) is much less than intraspecific divergence (15.4%–16.1%) |
| AB366479.1 *Acheilognathus argenteus* | Shallow interspecific divergence with *Acheilognathus tonkinensis* (0.1–3.1%) |
| KT591478.1 *Acanthorhodeus gracilis* | Shallow interspecific divergence with *Acheilognathus rhombeus* (0.2%–3%) |
| KC696535.1; KC696534.1 *Acrossocheilus yunnanensis* | Interspecific with *Acrossocheilus iridescens* (1.4%) is much less than intraspecific divergence (14.6%) |
| AF051857.1 *Acrossocheilus yunnanensis* | Deep intraspecific divergence (11.6%) |
| JQ346138.1 *Anematichthys apogon* | Interspecific divergence with *Anematichthys armatus* (0.3%) is much less than intraspecific divergence (10.9%) |
| FJ913813.1; EU082510.1 *Agosiachrys ogaste* | Deep intraspecific divergence (10.8%–11.6%) |
| AY281267.1 *Barbatula barbatula* | Deep intraspecific divergence (21.4%–22%) |
| AY004736.1; AY004745.1 *Barbus pallaryi* | Interspecific divergence with *Barbus antinorii* (0.1%) is much less than intraspecific divergence (7.6%) |
| JF798260.1; JF798259.1 *Barbus labiosa* | Interspecific divergence with *Luciobarbus rifensis* (0–0.5%) is much less than intraspecific divergence (3.4%–4.0%) |
| KT958256.1 *Barbus paludinosus* | Deep intraspecific divergence (21.3%) |
| AF180871.1 *Barbus zaphiri* | Shallow interspecific divergence with *Labeobarbus intermedius* (0.4%–1.4%) |
| AF287425.1; AF287424.1 *Barbus caninus* | Interspecific divergence with *Barbus balcanicus* (0.2%–1.1%) is much less than intraspecific divergence (8.4%) |
| AF145932.1*Barbus capito pectoralis* | Interspecific divergence with *Barbus graecus* (0.3%) is much less than intraspecific divergence (5.5%–5.6%) |
| AF180829.1; AF287421.1 *Barbus bynni occidentalis* | Interspecific divergence with *Barbus petitjeani* (0.1%–2.6%) is much less than intraspecific divergence (8.2%–8.7%) |
| KP659415.1 *Barbus holotaenia* | Deep intraspecific divergence (10.9%) |
| AF180854.1 *Barbus paytonii* | Shallow interspecific divergence with *Barbus fritschii* (0.1%–1.4%) |
| AF145928.1 *Barbus issenensis* | Shallow interspecific divergence with *Barbus massaensis* (0.1%–0.4%) |
| AY004738.1 *Barbus ksibi* | Shallow interspecific divergence with *Barbus magniatlanti* (0.5%–1.1%) |
| KU257525.1–KU257528 *Luciobarbus nasus* | Deep intraspecific divergence (9.5%–10.8%) |
| AF145927.1 *Barbus pallaryi* | Interspecific divergence with *Barbus lepineyi* (0.2%–1.3%) is much less than intraspecific divergence (7.6%) |
| GQ302799.1–GQ302803 *Barbus rebeli* | Interspecific divergence with *Barbus peloponnesius* (0.3%–0.4%) is much less than intraspecific divergence (5.1%–6.1%) |
| AY331026.1; AY331025.1 *Barbus borysthenicus* | Shallow interspecific divergence with *Barbus barbus* (0.4%–1.1%) |
| GU477596.1 *Barbus petenyi* | Deep intraspecific divergence (23.4%–23.9%) |
| AF112127.1 *Barbus petenyi* | Interspecific divergence with *Barbus carpathicus* (0.1%) is much less than intraspecific divergence (9.5%–10.3%) |
| AF287440.1; AF287439.1 *Barbus petenyi* | Interspecific divergence with *Barbus balcanicus* (0.1%–1.8%) is much less than intraspecific divergence (10.6%–11.2%) |
| AY004733.1 *Barbus labiosa* | Interspecific divergence with *Luciobarbus maghrebensis* (0.1%–0.2%) is much less than intraspecific divergence (3.5%–4%) |
| JQ346146.1 *Neolissochilus stracheyi* | Interspecific divergence with *Barbus wynaadensis* (0.7%) is much less than intraspecific divergence (5.9%) |
| NC_015533.1 *Barilius bendelisis* | Deep intraspecific divergence (32.6%) |
| KX060617.1 *Beaufortia kweichowensis* | Deep intraspecific divergence (18%) |
| AY952987.1 *Belligobio nummifer* | Interspecific divergence with *Hemibarbus medius* (2.2%–3.5%) is much less than intraspecific divergence (5.5%) |
| AB499046.1 *Biwia zezera* | Interspecific divergence with *Biwia yodoensis* (0–0.4%) is much less than intraspecific divergence (8.9%–10.1%) |
| AY281262.1 *Botia banarescui* | Shallow interspecific divergence with *Parabotia fasciata* (0.4%–3.1%) |
| HQ608621.1 *Campostoma pullum* | Shallow interspecific divergence with *Campostoma anomalum* (0.3%–1.7%) |
| DQ486813.1; DQ486805.1; DQ486827.1; DQ486826.1 *Campostoma anomalum* | Interspecific divergence with *Campostoma oligolepis* (0.3%–1.7%) is much less than intraspecific divergence (8.5%–9.3%) |
| DQ486795.1; DQ486807.1; DQ486806.1; DQ486819.1 *Campostoma anomalum* | Interspecific divergence with *Campostoma plumbeum* (2.6%–4.1%) is much less than intraspecific divergence (8.6%–9.1%) |
| DQ486792.1 *Campostoma pauciradii* | Interspecific divergence with *Campostoma anomalum* (0.4%–1.3%) is much less than intraspecific divergence (9.3%) |
| DQ486793.1; DQ486794.1; DQ486802.1; DQ486812.1; DQ486814.1; DQ486815.1; DQ486835.1; DQ486836.1; DQ486838.1 *Campostoma oligolepis* | Deep intraspecific divergence (10.2%–11.4%) |
| GU573943.1 *Candidia barbatus* | 100% identify with *Candidia pingtungensis* but deep intraspecific divergence (9.8%–10.3%) |
| GQ424021.1–GQ424024.1 *Capoeta antalyensis* | Interspecific divergence with *Capoeta baliki* (0.1%–2.3%) is much less than intraspecific divergence (3.5%–3.7%) |
| KC696558.1 *Carassioides cantonensis* | Shallow interspecific divergence with *Carassioides acuminatus* (0–0.9%) |
| GU135601.1 *Carassius auratus* | Interspecific divergence with *Carassius cuvieri* (0–0.7%) is much less than intraspecific divergence (6.8%–7.5%) |
| AY714387.1 *Carassius carassius* | Interspecific divergence with *Carassius auratus* (0%–0.3%) is much less than intraspecific divergence (8.5%–9.9%) |
| EU668752.1–EU668761.1; EU668783.1; EU668784.1; EU668786.1; EU668873.1–EU668880.1; KJ441242.1 *Catostomus plebeius* | Interspecific divergence with *Catostomus nebuliferus* (0.8%–2.3%) is much less than intraspecific divergence (8.4%–8.9%) |
| JX488793.1; JX488792.1 *Catostomus microps* | Interspecific divergence with *Catostomus occidentalis* (0.8%–2.9%) is much less than intraspecific divergence (11.3%) |
| JX488802.1; JX488801.1 *Catostomus platyrhynchus* | Interspecific divergence with *Catostomus virescens* (0.1%–0.3%) is much less than intraspecific divergence (2.8%–3.5%) |
| KJ441248.1 *Catostomus platyrhynchus* | Interspecific divergence with *Catostomus discobolus* (0.8%–1.8%) is much less than intraspecific divergence (2.9%–3.6%) |
| KJ441236.1 *Catostomus discobolus* | Interspecific divergence with *Catostomus plebeius* (0.8%–1.5%) is much less than intraspecific divergence (6.1%–7.0%) |
| JF799438.1 *Catostomus conchos* | Shallow interspecific divergence with *Catostomus bernardini* (0–2.1%) |
| JQ087872.1 *Catla catla* | Interspecific divergence with *Labeo rohita* (0.4%) is much less than intraspecific divergence (7.1%) |
| JF799432.1; JF799433.1 *Carpiodes cyprinus* | Interspecific divergence with *Carpiodes carpio* (1.3%–1.9%) is much less than intraspecific divergence (7.1%–7.0%) |
| KC701385.1 *Chanodichthys mongolicus* | Interspecific divergence with *Culter alburnus* (1.7%–1.8%) is much less than intraspecific divergence (5.6%–5.8%) |
| AF045986.1 *Chondrostoma lusitanicum* | Interspecific divergence with *Chondrostoma almacai* (0.2% ) is much less than intraspecific divergence (5.6%–6.0%) |
| AF045985.1 *Chondrostoma toxostoma* | Interspecific divergence with *Chondrostoma miegii* (0.9%) is much less than intraspecific divergence (2.3%–2.4%) |
| EU045786.1–EU045789.1 *Chondrostoma polylepis* | Interspecific divergence with *Chondrostoma duriense* (0.5%–1.0%) is much less than intraspecific divergence (4.5%–4.8%) |
| AY608650.1–AY608651.1; HQ235772.1 *Chuanchia labiosa* | Interspecific divergence with *Platypharodon extremus* (0–0.9%) is much less than intraspecific divergence (6.9%–7.3%) |
| JQ082362.1 *Chuanchia labiosa* | Interspecific divergence with *Gymnocypris przewalskii* (0.8%–1.3%) is much less than intraspecific divergence (7.5% –7.8%) |
| AY589501.1; AY608657.1–AY608663.1 *Platypharodon extremus* | Interspecific divergence with *Schizopygopsis pylzovi* (0–1.0%) is much less than intraspecific divergence (6.8%–7.3%) |
| KT005604.1–KT005606.1 *Paracanthocobitis mooreh* | Deep intraspecific divergence (18.0%–18.5%) |
| JF799438.1 *Catostomus conchos* | Shallow interspecific divergence with *Catostomus leopoldi* (0.3%–1.0%) |
| HM224260.1*Chelaethiops bibie* | Deep intraspecific divergence (12.4%) |
| KF574666.1–KF574667.1; KF574672.1–KF574678.1; KF574680.1 *Cirrhinus reba* | Interspecific divergence with *Bangana ariza* (0–0.3%) is much less than intraspecific divergence (4.4%–5.0%) |
| AY463100.1 *Cirrhinus mrigala* | Deep intraspecific divergence (11.7%–12.1%) |
| KX289615.1 *Claea dabryi* | Deep intraspecific divergence (25.2%) |
| AB860297.1; DQ105231.1; JX888906.1; KP133112.1; JN858879.1–JN858894.1 *Cobitis lutheri* | Deep intraspecific divergence (13.7%–14.4%) |
| AY191580.1 *Cobitis punctilineata* | Interspecific divergence with *Cobitis strumicae* (0.3%–0.7%) is much less than intraspecific divergence (4.3%) |
| AM910634.1–AM910639.1; AM711121.1–AM711124.1; AY281266.1; AY625699.1; JX888902.1 *Cobitis sinensis* | Deep intraspecific divergence (11.7%–17.2%) |
| DQ217374.1 *Cobitis turcica* | Interspecific divergence with *Cobitis strumicae* (0%–1.8%) is much less than intraspecific divergence (10.2%–11.9%) |
| KJ755229.1–KJ755238.1; EU082516.1; EU082515.1 *Codoma ornata* | Deep intraspecific divergence (9.6%–10.5%) |
| HQ235761.1 *Cosmochilus cardinalis* | Interspecific divergence with *Cosmochilus harmandi* (0–0.1% ) is much less than intraspecific divergence (12.5%) |
| AY392468.1–AY392471.1 *Crossostoma fascicauda* | Interspecific divergence with *Crossostoma stigmata* (0.1%–0.5%) is much less than intraspecific divergence (6.9%–7.4%) |
| JX083158.1 *Crossocheilus diplochilus* | Shallow interspecific divergence with *Crossocheilus latius* (1.0%–1.2%) |
| KF760463.1 *Culter brevicauda* | Shallow interspecific divergence with *Culter alburnu*s (0.3%–0.5%) |
| KF029689.1 *Culter erythropterus* | Interspecific divergence with *Culter alburnus* (0–1.7%) is much less than intraspecific divergence (9.1%) |
| GQ275184.1–GQ275188.1; KR061540.1; KR061545.1–KR061547.1; KR061550.1; KR061551.1; KR061559.1; KR061562.1; KR061563.1 *Cyprinella lutrensis* | Deep intraspecific divergence (10.2%–14.7%) |
| GQ275177.1; GQ275178.1; KR061629.1–KR061633.1 *Cyprinella lepida* | Deep intraspecific divergence (12.6%–13.2%) |
| GQ275232.1–GQ275233.1 *Cyprinella whipplei* | Interspecific divergence with *Cyprinella spiloptera* (1.9%–2.9%) is much less than intraspecific divergence (16.5%–16.1%) |
| KR061638.1–KR061639.1 *Cyprinella venusta* | Deep intraspecific divergence (9.8%–11.4%) |
| JX488770.1 *Cycleptus meridionalis* | Shallow interspecific divergence with *Cycleptus elongatus* (0–2.6%) |
| HM536798.1; HQ235758.1; KR869145.1 *Cyprinus multitaeniata* | Interspecific divergence with *Cyprinus carpio* (1.0%–1.1%) is much less than intraspecific divergenc (7.8%–8.6%) |
| JX042203.1 *Cyprinus pellegrini* | Shallow interspecific divergence with *Cyprinus carpio* (1.4%–1.8%) |
| KR869144.1 *Cyprinus acutidorsalis* | Shallow interspecific divergence with *Cyprinus carpio* (0.7%–1.2%) |
| KR606519.1 *Danio nigrofasciatus* | Interspecific divergence with *Danio rerio* (0.1%–7.8%) is much less than intraspecific divergence (21.2%) |
| NC_015525.1 *Danio dangila* | Deep intraspecific divergence (13.4%) |
| KP407138.1 *Devario chrysotaeniatus* | Deep intraspecific divergence (22.5%) |
| DQ324077.1–DQ324079.1; JN812368.1–JN812373.1; EU082490.1 *Dionda episcopa* | Deep intraspecific divergence (12.9%–14.6%) |
| EU082501.1–EU082503.1 *Dionda nigrotaeniata* | Interspecific divergence with *Dionda flavipinnis* (0.1%–6.5%) is much less than intraspecific divergence (14.6% –18.6%) |
| AF374407.1 *Distoechodon compressus* | Shallow interspecific divergence with *Distoechodon tumirostris* (0.2%–3.6%) |
| KJ669372.1 *Discogobio tetrabarbatus* | Interspecific divergence with *Discogobio bismargaritus* (0.3%–2.2%) is much less than intraspecific divergence (7.9%–8.1%) |
| KJ997760.1 *Discogobio yunnanensis* | Interspecific divergence with *Discogobio bismargaritus* (0.2%–2.3%) is much less than intraspecific divergence (6.1%) |
| EF151101.1 *Esomus metallicus* | Deep intraspecific divergence (12.5%–12.9%) |
| KC763688.1 *Ericymba buccata* | Deep intraspecific divergence (16.8%) |
| NC_031571.1 *Erimystax dissimilis* | Interspecific divergence with *Exoglossum maxillingua* (0.1%–0.2%) is much less than intraspecific divergence (17.6%–18.0%) |
| JX074279.1 *Garra ornata* | Shallow interspecific divergence with *Garra congoensis* (1.1%–2.8%) |
| JX074243.1 *Garra kempi* | Deep intraspecific divergence (10.0%) |
| KC696551.1 *Garra pingi pingi* | Shallow interspecific divergence with *Garra imberba* (0–3.0%) |
| JQ864580.1 *Garra bourreti* | Shallow interspecific divergence with *Garra orientalis* (0.2%–1.1%) |
| AY281272.1 *Gastromyzon ctenocephalus* | Deep intraspecific divergence (10.3%) |
| KU314697.1 *Gobiobotia pappenheimi* | Shallow interspecific divergence with *Gobiobotia naktongensis* (0.9%–3.6%) |
| FJ913816.1 *Gila robusta* | Interspecific divergence with *Gila minacae* (0–1.7%) is much less than intraspecific divergence (7.6%–7.9%) |
| KF514254.1 *Gila robusta* | Interspecific divergence with *Gila atraria* (0.8%–1.1%) is much less than intraspecific divergence (4.7%–7.6%) |
| LC098154.1–LC098157.1; AB677435.1–AB677438.1 *Gnathopogon elongatus* | Interspecific divergence with *Gnathopogon caerulescens* (3.1%–3.7%) is much less than intraspecific divergence (7.4%––8.6%) |
| HQ198884.1 *Gymnocypris dobula* | Shallow interspecific divergence with *Gymnocypris namensis* (0.7%–1.1%) |
| DQ058285.1–DQ058290.1 *Gymnocypris eckloni* | Interspecific divergence with *Schizopygopsis pylzovi* (0.3%–0.7%) is much less than intraspecific divergence (7.1%–7.8%) |
| DQ058291.1 *Gymnocypris scoliostomus* | Shallow interspecific divergence with *Gymnocypris eckloni* (0–1.0%) |
| FJ534394.1–FJ534403.1 *Gymnocypris eckloni eckloni* | Interspecific divergence with *Schizopygopsis pylzovi* (0%–0.7%) is much less than intraspecific divergence (6.9%–7.9%) |
| KT833086.1 *Gymnocypris waddelli* | Interspecific divergence with *Oxygymnocypris stewartii* (0–0.4%) is much less than intraspecific divergence (10.4%–11.0%) |
| KC734002.1 *Gymnocypris potanini* | Interspecific divergence with *Gymnocypris przewalskii* (2.7%–3.1%) is much less than intraspecific divergence (6.6%) |
| AY463499.1 *Gymnocypris potanini* | Interspecific divergence with *Schizopygopsis anteroventris* (0.3%–1.5%) is much less than intraspecific divergence (9.1%) |
| KJ081401.1–KJ081422.1; AY463513.1 *Gymnodiptychus dybowskii* | Deep intraspecific divergence (11.1%–11.7%) |
| KT833112.1 *Gymnodiptychus pachycheilus* | Interspecific divergence with *Chizopygopsis anteroventris* (0–0.1%) is much less than intraspecific divergence (15.9%–16.0%) |
| KT751364.1 *Hemibarbus labeo* | Interspecific divergence with *Semilabeo notabilis* (0–0.4%) is much less than intraspecific divergence (19.1%–19.4%) |
| KT833108.1; KT833082.1 *Herzensteinia microcephalus* | Interspecific divergence with *Schizopygopsis younghusbandi* (0.1%–0.4%) is much less than intraspecific divergence (4.7%–5.4%) |
| KT833110.1 *Herzensteinia microcephalus* | Interspecific divergence with *Platypharodon extremus* (0.1–0.9%) is much less than intraspecific divergence (6.8%–7.1%) |
| KT361083.1 *Hemiculter bleekeri bleekeri* | Shallow interspecific divergence with *Hemiculter leucisculus* (0.5%–1.3%) |
| KF021222.1 *Hemiculter leucisculu* | Deep intraspecific divergence (12.7%–14.9%) |
| KF020986.1–KF020988.1; KF021182.1–KF021209.1; KF021211.1–KF021216.1 *Hemiculter leucisculus* | Interspecific divergence with *Hemiculter bleekeri* (0.4%–1.2%) is much less than intraspecific divergence (10.8%–13.6%) |
| KF760461.1 *Hemiculter eigenmanni* | Interspecific divergence with *Hemiculter bleekeri* (0.8%) is much less than intraspecific divergence (11.2%–11.5%) |
| KF029695.1 *Hemiculter lucidus* | Shallow interspecific divergence with *Hemiculter bleekeri* (1.2%–1.7%) |
| KU314694.1 *Huigobio chenhsienensis* | Interspecific divergence with *Platysmacheilus longibarbatus* (3.4%) is much less than intraspecific divergence (8.6%–8.7%) |
| KC984317.1–KC984319.1 *Huigobio chenhsienensis* | Interspecific divergence with *Microphysogobio tafangensis* (0.7%–0.8%) is much less than intraspecific divergence (8.7%–8.8%) |
| JQ231114.1 *Hypophthalmichthys molitrix* | Interspecific divergence with *Hypophthalmichthys nobilis* (0.2%–0.5%) is much less than intraspecific divergence (7.6%–8.8%) |
| HM010724.1 *Hypselobarbus lithopidos* | Interspecific divergence with *Hypselobarbus pulchellus* (0.1%) is much less than intraspecific divergence (6.8%) |
| KC524523.1; KC524522.1 *Iksookimia hugowolfeldi* | Interspecific divergence with *Iksookimia yongdokensis* (4.1%) is much less than intraspecific divergence (6.8%) |
| Z75909.1 *L.carolitertii* | Interspecific divergence with *Squalius pyrenaicus* (1.5%–1.7%) is much less than intraspecific divergence (5.6%–6.3%) |
| Z75921.1; Z75922.1; Z75918.1; Z75919.1 *L.pyrenaicus* | Interspecific divergence with *Leuciscus carolitertii* (5.0%–5.7%) is much less than intraspecific divergence (7.2%–8.9%) |
| AY509827.1 *Leuciscus cephalus* | Interspecific divergence with *Squalius cephalus* (0.1%–0.5%) is much less than intraspecific divergence (4.6%–5.5%) |
| AF421792.1; AJ252803.1; AJ252785.1; AJ252798.1; AJ252789.1 *Leuciscus cephalus* | Interspecific divergence with *Squalius squalus* (0.8%–2.3%) is much less than intraspecific divergence (4.5%–7.8%) |
| AJ252783.1 *Leuciscus cephalus* | Deep intraspecific divergence (10.3%–13.5%) |
| AF421803.1; AF421801.1; AJ252791.1 *Leuciscus cephalus* | Interspecific divergence with *Squalius laietanus* (0–0.5 %) is much less than intraspecific divergence (4.2%–7.2%) |
| AJ252818.1; AJ252819.1 *Leuciscus lucumonis* | Interspecific divergence with *Squalius squalus* (0–1.7%) is much less than intraspecific divergence (7.9%–8.6%) |
| JX074260.1 *Labeo bata* | Interspecific divergence with *Labeo boggut* (0.2%–0.4%) is much less than intraspecific divergence (8.9%–9.3%) |
| KP025676.1 *Labeo fimbriatus* | Interspecific divergence with *Labeo gonius* (0–1.8%) is much less than intraspecific divergence (7.8%–8.3%) |
| HQ235751.1 *Labeo forskalii* | Interspecific divergence with *Epalzeorhynchos bicolor* (0.1%) is much less than intraspecific divergence (15.9%–16.1%) |
| AY463099.1 *Labeo rohita* | Interspecific divergence with *Cirrhinus mrigala* (0.1%–0.4%) is much less than intraspecific divergence (11.9%–12.1%) |
| KU577402.1 *Labeo gonius* | Interspecific divergence with *Labeo rohita* (0.1%–0.5% )is much less than intraspecific divergence ( 9.2%–9.7%) |
| KC696529.1; KC631291.1 *Labeo pierrei* | Interspecific divergence with *Labeo yunnanensis* (0.4%–0.5%) is much less than intraspecific divergence (15.1%–15.6%) |
| KC631289.1 *Labeo barbatulus* | Shallow interspecific divergence with *Catla catla* (0–0.2%) |
| AJ251091.1 *Ladigesocypris ghigii* | 100% identify with *Ladigesocypris irideus* but deep intraspecific divergence (12.2%) |
| JX443003.1 *Lavinia exilicaud* | Shallow interspecific divergence with *Hesperoleucus symmetricus* (0.7%–2.1%) |
| KC631292.1 *Labiobarbus lineatus* | Interspecific divergence with *Labiobarbus leptocheilus* (0.3%–0.5%) is much less than intraspecific divergence (7.9%–8.3%) |
| KC865424.1 *Leptobotia microphthalma* | Interspecific divergence with with *Leptobotia elongata* (0.1%–0.3%) is much less than intraspecific divergence (6%–6.2%) |
| DQ664303.1; DQ664305.1 *Leuciscus leuciscus* | Interspecific divergence with *Leuciscus burdigalensis* (1.1%–3.3%) is much less than intraspecific divergence (4.8%––6.7%) |
| JN176993.1; GU084226.1–GU084244.1; GU084246.1 *Lepturichthys fimbriata* | Interspecific divergence with *Lepturichthys dolichopteru* (0.2%–1.3%) is much less than intraspecific divergence (7.4%––8.3%) |
| HM224293.1 *Leptocypris niloticus* | Deep intraspecific divergence (13.9%) |
| KU257525.1–KU257528.1 *Luciobarbus nasu* | Deep intraspecific divergence (9.5%–10%) |
| KU257538.1–KU257539.1 *Luciobarbusnasus* | Interspecific divergence with *Luciobarbusksibi* (0–0.7%) is much less than intraspecific divergence (5.8%–6.2%) |
| NC018816.1 *Megalobrama terminalis* | Interspecific divergence with *Parabramis pekinensis* (0.2%–0.4%) is much less than intraspecific divergence (9.4%) |
| HM224304.1 *Metzia formosae* | Interspecific divergence with *Metzia longinasus* (1.7%) is much less than intraspecific divergence (10.2%) |
| KF997093.1 *Metzia mesembrinum* | Interspecific divergence with *Metzia lineata* (0.1–0.4%) is much less than intraspecific divergence (3.2%) |
| JN177000.1; KM077128.1 *Metahomaloptera omeiensis* | Deep intraspecific divergence (9.4%–19.4%) |
| AY953013.1 *Microphysogobio tungtingensis* | Shallow interspecific divergence with *Microphysogobio fukiensis* (1.7%–1.8%) |
| EU934489.1 *Microphysogobio elongatus* | Interspecific divergence with *Microphysogobio fukiensis* (1.5%–7.0%) is much less than intraspecific divergence (10.0%) |
| KT075099.1 *Microphysogobio tafangensis* | Deep intraspecific divergence (16.1%) |
| AB473261.1–AB473263.1; AB473275.1–AB4732877.1; AB473308.1–AB473309.1; AB614359.1; DQ026434.1; AB473312.1 –AB473314.1.1 *Misgurnus anguillicaudatus* | Deep intraspecific divergence (18.4%–19.4%) |
| KF736233.1; DQ105238.1 *Misgurnus anguillicaudatus* | Interspecific divergence with *Misgurnus bipartitus* (1.1%–2.9%) is much less than intraspecific divergence (6.2%–7.3% ) |
| JN858850.1–JN858853.1 *Misgurnus mohoity* | Interspecific divergence with *Misgurnus bipartitus* (0.4%–2.7%) is much less than intraspecific divergence (19.2%–19.5%) |
| HQ235762.1 *Mystacoleucus marginatus* | Interspecific divergence with *Mystacoleucus lepturus* (0.3%–0.5%) is much less than intraspecific divergence (8.8%) |
| EF151105.1 *Microrasbora kubotai* | Interspecific divergence with *Microdevario kubotai* (0.3%) is much less than intraspecific divergence (6.2%) |
| JF799498.1; JF799497.1; JF799494.1 *Moxostoma congestum* | Interspecific divergence with *Moxostoma albidum* (0–1.1%) is much less than intraspecific divergence (10.7% –11.3) |
| KP256264.1–KP256270.1 *Moxostoma austrinum* | Interspecific divergence with *Moxostoma albidum* (0–1.1%) is much less than intraspecific divergence (5.1%–7.5%) |
| JX488820.1 *Moxostoma carinatum* | Interspecific divergence with *Moxostoma valenciennesi (*0.3%–0.4%) is much less than intraspecific divergence (8.7%–9.1%) |
| JN858856.1–JN858859.1 *Misgurnus nikolskyi* | Deep intraspecific divergence (10.4%–12.9%) |
| JF799520.1; JF799521.1 *Moxostoma mascotae* | Shallow interspecific divergence with *Moxostoma austrinum* (0.1%–2.6%) |
| AF454889.1 *Moxostoma pisolabrum* | Shallow interspecific divergence with *Moxostoma macrolepidotum* (0.3%–2%) |
| KT834522.1 *Notropis percobromus* | Interspecific divergence with *Notropis rubellus* (0.3%–1.1%) is much less than intraspecific divergence (5.9%–6.5%) |
| EU084862.1–EU084867.1 *Notropis rubellus* | Interspecific divergence with *Notropis suttkusi* (5.4%–6.3%) is much less than intraspecific divergence (8.1%–10.8%) |
| AF352276.1 *Notropis girardi* | Shallow interspecific divergence with *Notropis wickliffi* (0.9–1.4%) |
| AF469142.1; AF469141.1 *Notropis calientis* | Interspecific divergence with *Notropis grandis* (0.2%–0.9%) is much less than intraspecific divergence (6.1%–6.6%) |
| AF352268.1 *Notropis volucellus* | 100% identify with *Notropis uranoscopus* but deep intraspecific divergence (15.9%–16.6%) |
| KC696525.1 *Neolissochilus hexagonolepis* | Interspecific divergence with *Neolissochilus stracheyi* (1.4%–1.6%) is much less than intraspecific divergence (3.8%–4.9%) |
| HM536823.1 *Neolissochilus stracheyi* | Interspecific divergence with *Neolissochilus hexagonolepis* (0–2.6%) is much less than intraspecific divergence (5.5%–5.9%) |
| AY646603.1–AY646609.1; AY646620.1–AY646649.1; FJ601934.1–FJ601940.1; FJ601944.1–FJ601947.1; FJ601958.1–FJ601966.1; FJ601970.1; FJ601976.1–FJ601980.1; FJ601988.1–FJ601992.1; FJ601999.1; FJ602004.1 *Opsariichthys bidens* | Interspecific divergence with *Opsariichthys hainanensis* (0.1–8.1%) is much less than intraspecific divergence (13.9%–14.8%) |
| HQ235764.1 *Onychostoma rara* | Interspecific divergence with *Acrossocheilus monticola* (0–0.8%) is much less than intraspecific divergence (7.6%) |
| KF021233.1 *Onychostoma simum* | Interspecific divergence with *Onychostoma gerlachi* (0.1%) is much less than intraspecific divergence (14%–14.1%) |
| KC696547.1 *Onychostoma gerlachi* | Interspecific divergence with *Onychostoma lepturum* (0.7%–2.1%) is much less than intraspecific divergence (10.1%) |
| KC696548.1*Onychostoma gerlachi* | Interspecific divergence with *Onychostoma meridionale* (3.3%–5.3%) is much less than intraspecific divergence (7.9%) |
| KJ940961.1; KJ940960.1 *Opsariichthys minutus* | Shallow interspecific divergence with *Opsariichthys bidens* (0.3%–1.5%) |
| GQ275152.1; AF261221.1 *Opsopoeodus emiliae* | Deep intraspecific divergence (11.3%–12.4%) |
| EU670348.1; EU670347.1 *Pangio anguillaris* | Deep intraspecific divergence (9.7%–10.6%) |
| EU670358.1 *Pangio cuneovirgata* | Deep intraspecific divergence (9.7%–10.4%) |
| EU670363.1 *Pangio doriae* | Deep intraspecific divergence (24.6%–25.3%) |
| GQ174323.1; GQ174324.1 *Pangio oblonga* | Deep intraspecific divergence (10.8%–11.7%) |
| LC167412.1 *Paracobitis malapterura* | Deep intraspecific divergence (22%–22.4%) |
| KP659417.1 *Pethia phutunio* | Deep intraspecific divergence (13.8%) |
| KC696520.1 *Pethia ticto* | Interspecific divergence with *Puntius ticto* (16.7%–16.9%) is much less than intraspecific divergence (0.1%–0.5%) |
| KP861804.1; KP861803.1 *Pethia ticto* | Deep intraspecific divergence (16.9%–17.5%) |
| KM186183.1; KF771003.1 *Paramisgurnus dabryanu* | Deep intraspecific divergence (18.1%–19.6%) |
| EF094550.1 *Phoxinus phoxinus* | Deep intraspecific divergence (9.2%–11.4%) |
| GQ275159.1; GQ275158.1*Pimephales promelas* | Deep intraspecific divergence (15.2%–15.9%) |
| AB236729.1 *Phoxinus steindachneri* | Deep intraspecific divergence (11.9%–12.2%) |
| HM560130.1; HM560129.1 *Pseudophoxinus anatolicus* | Interspecific divergence with *Pseudophoxinus crassus* (0.7%–1.1%) is much less than intraspecific divergence (11.4%–11.5%) |
| HQ167617.1 *Pseudophoxinus kervillei* | Interspecific divergence with *Pseudophoxinus zekayi* (1%–1.1%) is much less than intraspecific divergence (15.1%) |
| NC_031574.1 *Pseudochondrostoma polylepis* | Shallow interspecific divergence with *Chondrostoma polylepis* (0.8%–1%) |
| KU314692.1 *Platysmacheilus longibarbatus* | Deep intraspecific divergence (15.1%) |
| KC567006.1 *Poropuntius huangchuchieni* | Interspecific divergence with *Poropuntius opisthoptera* (0.3%–0.4%) is much less than intraspecific divergence (4.1%–5%) |
| KT182474.1 *Pseudaspius leptocephalus* | Deep intraspecific divergence (31.9%–32.2%) |
| KT335798.1 *Pteronotropis euryzonus* | Interspecific divergence with *Pteronotropis grandipinnis* (0.9%), is much less than intraspecific divergence (5.4%–5.8%) |
| JX470431.1; JX470430.1 *Puntius denisonii* | Deep intraspecific divergence (12.3%–13.1%) |
| KF019637.1 *Puntius denisonii* | Interspecific divergence with *Sahyadria denisonii* (0.4%) is much less than intraspecific divergence (7.0%–7.2%) |
| EU241456.1 *Puntius gelius* | Deep intraspecific divergence (23.5%–24%) |
| KX101229.1 *Pseudogastromyzon fasciatus* | Interspecific divergence with P*seudogastromyzon tungpeiensis* (0.8%) is much less than intraspecific divergence (16.3%) |
| AY882863.1–AY882888.1; JN003316.1; KM999932.1; KU314695.1 *Pseudogobio vaillanti* | Interspecific divergence with with *Pseudogobio esocinus* (0.3%–9.1%) is much less than intraspecific divergence (9.2%–11.4%) |
| AY882889.1; AY882901.1 *Pseudogobio vaillanti* | Deep intraspecific divergence (9.3%–11.3%) |
| NC_031632.1 *Psilorhynchus balitora* | Deep intraspecific divergence (22.2%–22.8%) |
| AB677451.1; AB677450.1 *Pseudorasbora pumila* | Interspecific divergence with *Pseudorasbora pugnax* (0–1.0%) is much less than intraspecific divergence (8.1% –9.1%) |
| KM207644.1 *Psilorhynchus balitora* | Interspecific divergence with *Psilorhynchus homaloptera* (1.3%) is less than the intraspecific divergence (2.1%–2.3%) |
| KT633641.1 *Ptychidio macrops* | Shallow interspecific divergence with *Ptychidio jordani* (0–1.1%) |
| KM364664.1 *Ptychobarbus kaznakovi* | Interspecific divergence with *Ptychobarbus dipogon* (0.5%–1.1%) is much less than intraspecific divergence (7.8%–8.2%) |
| KT215538.1 *Ptychobarbus kaznakovi* | Deep intraspecific divergence (19.8%–21.4%) |
| HM010724.1 *Hypselobarbus lithopidos* | 100% identify with *Puntius jerdoni* but deep intraspecific divergence (6.8%) |
| AF051875.1 *Raiamas guttatus* | Deep intraspecific divergence (26.4%) |
| JQ346143.1 *Raiamas guttatus* | Deep intraspecific divergence (15.7%–16.0%) |
| HM224345.1 *Rasbora daniconius* | Deep intraspecific divergence (26.9–27.4%) |
| KT267196.1 *Rhinichthys osculus* | Deep intraspecific divergence (26.9%–27.3%) |
| AF452078.1; KF640094.1; JX442984.1 *Rhinichthys atratulus* | Interspecific divergence with *Rhinichthys obtusus* (0–3.6%) is much less than intraspecific divergence (6.6–8.2%) |
| KJ641843.1 *Rhynchocypris lagowskii* | Deep intraspecific divergence (11.2%–11.4%) |
| KF734881.1 *Rhynchocypris lagowskii* | Interspecific divergence with *Phoxinus semotilus* (4.7%) is much less than intraspecific divergence (7.7%–7.8%) |
| AY952992.1 *Rhinogobio cylindricus* | Interspecific divergence with *Rhinogobio ventralis* (0.3%) is much less than intraspecific divergence (11.4%) |
| AF045988.1; AF045987.1 *Rutilus lemmingii* | Shallow divergence with *Chondrostoma lemmingii* (0.3%–1.1%), the same species but named differently |
| KJ631750.1 *Rhinogobio typus* | Interspecific divergence with *Rhinogobio ventralis* (0.2%) is much less than intraspecific divergence (3.4%–4.0%) |
| AF051876.1; KT004415.1 *Rhodeus ocellatus* | Deep intraspecific divergence (20.0%–20.6%) |
| KF533721.1; KF410794.1; AB366522.1 *Rhodeus sinensis* | Deep intraspecific divergence (12.6%–19.6%) |
| KF980890.1; AB366499.1 *Rhodeus fangi* | Interspecific divergence with *Rhodeus notatus* (0.9%–1.5%) is much less than intraspecific divergence (3.9%–4.5%) |
| AB366525.1; AB366523.1 *Rhodeus spinalis* | Deep intraspecific divergence (13.6%–14.2%) |
| FJ025083.1 *Rutilus basak* | Interspecific divergence with *Rutilus prespensis* (0.2%–0.7%) is much less than intraspecific divergence (4.2–4.3%) |
| FJ025073.1 *Rutilus vegariticus* | Shallow interspecific divergence with *Rutilus rutilus* (0–5.5%) |
| FJ025076.1; FJ025064.1; FJ025075.1; FJ025065.1 *Rutilus pigus* | 100% identify with *Rutilus virgo* but deep intraspecific divergence (8.5%–9.2%) |
| AF045989.1 *Rutilus lemmingii* | Interspecific divergence with *Chondrostoma oretanum* (0.2%–0.8%) is much less than intraspecific divergence (3.6–3.7%) |
| FJ196822.1 *Rectoris posehensis* | Interspecific divergence with *Sinocrossocheilus bamaensis* (0–0.1%) is much less than intraspecific divergence (11.6%) |
| AF499188.1 *Sabanejewia bulgarica* | Shallow interspecific divergence with *Sabanejewia balcanica* (0.2–1.3%) |
| NC_031545.1 *Sabanejewia radnensis* | Shallow interspecific divergence with *Sabanejewia balcanica* (0.4–1.8%) |
| KF534790.1 *Saurogobio dabryi* | Interspecific divergence with *Romanogobio tenuicorpu*s (0.2%–0.3%) is much less than intraspecific divergence (20.1%–20.4%) |
| AY952983.1; EF193462.1; EF193430.1–EF193432 .1 *Sarcocheilichthys nigripinnis* | Interspecific divergence with *Sarcocheilichthys czerskii* (0.1%–1%) is much less than intraspecific divergence (7.4%–7.8%) |
| JN003337.1 *Sarcocheilichthys soldatovi* | 100% identify with *Sarcocheilichthys czerskii* |
| JAF454901.1 *Scartomyzon congestus* | Deep intraspecific divergence (11.1%–13.3%) |
| KF534790.1; AY245091.1 *Saurogobio dabryi* | Deep intraspecific divergence (11.8%–20.1%) |
| AY625698.1 *Schistura longa* | Interspecific divergence with S*ectoria heterognathos* (0.5%) is much less than intraspecific divergence (20.0%) |
| KT034313.1–KT034321.1 ; KT833094.1 S*chizopyge lissolabiata* | Interspecific divergence with *Schizothorax yunnanensis* yunnanensis (0–0.5 %) is much less than intraspecific divergence (5.9%–6.0%) |
| KT034393.1–KT034401.1; AY954252.1 *Schizothorax yunnanensis yunnanensis* | Interspecific divergence with *Schizopyge lissolabiata* (0–1.1%) is much less than intraspecific divergence (5.4%–6.1%) |
| KP892531.1 *Schizothorax yunnanensis paoshanensis* | Interspecific divergence with *Schizopyge gongshanensis* (0.7%–1%) is much less than intraspecific divergence (5.7%–5.9%) |
| KT034238.1 *Schizopyge nukiangensis* | Shallow interspecific divergence with *Schizopyge gongshanensis* (0.1%–0.3%) |
| EU158043.1; EU158045.1; EU158046.1; EU158052.1 *Schizothorax lissolabiatus* | Interspecific divergence with *Schizothorax yunnanensis yunnanensis* (0–0.4%) is much less than intraspecific divergence (4.9%–5.5%) |
| EU158048.1 *Schizothorax lissolabiatus* | Interspecific divergence with *Schizothorax yunnanensis weiningensis* (0.5%) is much less than intraspecific divergence (6.2%–6.6%) |
| EU158027.1; EU158026.1; AY954253.1 *Schizothorax griseus* | Interspecific divergence with *Schizopyge lissolabiata* (0–1.8%) is much less than intraspecific divergence (5.9%–7.0%) |
| EU158030.1; EU158031.1 *Schizothorax griseus* | Interspecific divergence with *Schizothorax yunnanensis weiningensis* (0.5%) is much less than intraspecific divergence (6.2%–6.6%) |
| AY954286.1 *Schizothorax yunnanensis paoshanensis* | Interspecific divergence with *Schizothorax meridionalis* (0–1.1%) is much less than intraspecific divergence (6.4%–6.5%) |
| EU158025.1 *Schizothorax griseus* | Interspecific divergence with *Schizothorax meridionalis* (0.1%–1.1%) is much less than intraspecific divergence (5.6%–6.2%) |
| KT833090.1; KT833089.1 *Schizothorax waltoni* | Deep intraspecific divergence (19.2%–20.7%) |
| KF928796.1 *Schizothorax plagiostomus* | Interspecific divergence with *Schizothorax molesworthi* (0–0.3%) is much less than intraspecific divergence (10.6%–11.1%) |
| HQ198881.1 *Schizothorax wangchiachii* | Interspecific divergence with *Schizothorax oconnori* (0.3%–2.8%) is much less than intraspecific divergence (7.1%–7.9%) |
| JQ844133.1; AY954276.1 *Schizothorax biddulphi* | Interspecific divergence with *Aspiorhynchus laticeps* (0.3%) is much less than intraspecific divergence (8.4%–8.8%) |
| DQ309364.1; DQ646888.1 *Schizopygopsis kialingensis* | Interspecific divergence with *Schizopygopsis pylzovi* (1.5%–1.8%) is much less than intraspecific divergence (3.1%–3.3%) |
| DQ646898.1–DQ646900.1; DQ309361.1; DQ309360.1; JQ082357.1 *Schizopygopsis malacanthus* | Interspecific divergence with *Schizopygopsis kialingensis* (1.7%–2.0%) is much less than intraspecific divergence (8.2%–8.6%) |
| JQ082360.1 *Schizopygopsis malacanthus chengi* | Interspecific divergence with *Schizopygopsis pylzovi* (0.1%%–0.8%) is much less than intraspecific divergence (6.7%–7.2%) |
| KT268317.1; KM364663.1 *Schizopygopsis malacanthus* | Interspecific divergence with *Gymnocypris przewalskii* (0.5%–0.8%) is much less than intraspecific divergence (6.1%) |
| DQ309367.1; DQ646911.1; KC782632.1–KC782634.1 *Schizopygopsis thermalis* | Interspecific divergence with *Herzensteinia microcephalus* (0–3%) is much less than intraspecific divergence (5.3%–5.7%) |
| AY608654.1 –AY608656.1 *Schizopygopsis pylzovi* | Interspecific divergence with *Gymnocypris przewalskii* (0.1%–0.4%) is much less than intraspecific divergence (7.8%–8.3%) |
| GU589570.1 *Sinocyclocheilus furcodorsalis* | Interspecific divergence with *Sinocyclocheilus anatirostris* (2.5%) is much less than intraspecific divergence (8.7%) |
| HM536792.1 *Sinocyclocheilus macrophthalmus* | Interspecific divergence with *Sinocyclocheilus xunlensis* (0.7%–0.8%) is much less than intraspecific divergence (3.8%) |
| NC_031540.1 *Squalius cephalus* | Interspecific divergence with *Leuciscus cephalus* (0.8%–2.6%) is much less than intraspecific divergence (4.9%–5.2%) |
| EU856045.1; EU856046 *Squalius cephalus* | Interspecific divergence with *Squalius squalus* (0.4%–2%) is much less than intraspecific divergence (5.0%–5.7%) |
| DQ263227.1–DQ263233.1; AJ698710.1; JQ436542.1–JQ436545.1; DQ003238.1 *Squalius alburnoides* | Interspecific divergence with *Squalius pyrenaicus* (0.2%–2.6%) is much less than intraspecific divergence (11.5%–11.7%) |
| AJ251093.1 *Leuciscus zrmanjae* | 100% identify with *Squalius zrmanjae* but deep intraspecific divergence (8.7%) |
| AF452085.1; AF452084.1; JX443057.1; JX443058.1 *Snyderichthys copei* | Interspecific divergence with *Lepidomeda aliciae* (0–1.2%) is much less than intraspecific divergence (8.8%–9.1%) |
| KJ364658.1 *Squalidus gracili* | Deep intraspecific divergence (19.1%) |
| AY952986.1 *Squalidus nitens* | Shallow interspecific divergence with *Squalidus argentatus* (0.2%–2.4%) |
| EF151120.1 *Sundadanio axelrodi* | Deep intraspecific divergence (15.4%–16.8%) |
| GQ174365.1 *Syncrossus helodes* | Deep intraspecific divergence (14.7%–16%) |
| KC852197.1 *Spinibarbus denticulatus* | Interspecific divergence with *Spinibarbus caldwelli* (0.2%–2.8%) is much less than intraspecific divergence (12.4%–13.1%) |
| AB366533.1; AB366532.1 *Tanakia lanceolata* | Interspecific divergence with *Acheilognathus intermedia* (0.3%–0.7%) is much less than intraspecific divergence (10.6%–10.7%) |
| KF866141.1–KF866143.1 *Tanakia lanceolata* | Deep intraspecific divergence (10.1%–10.8%) |
| AB108957.1; AB108966.1; AB108967.1; AB108979.1 *Tanakia limbata* | Interspecific divergence with *Tanakia Lanceolata* (0.2–4.1%) is much less than intraspecific divergence (16.2%–17.7%) |
| KF866187.1; KF866191.1; KF866190.1 *Tanakia koreensis* | Interspecific divergence with *Acheilognathus somjinensis* (0.5%–0.7%) is much less than intraspecific divergence (10.6%–11%) |
| KF410805.1; AB366530. 1 *Tanakia koreensis* | Deep intraspecific divergence (10.6%–11%) |
| HM560209.1; HM560208.1 *Telestes turskyi* | Interspecific divergence with *Squalius microlepis* (0.8%–1.1%) is much less than intraspecific divergence (16.1%–16.3%) |
| JN188377.1 *Telestes karsticus* | Interspecific divergence with *Telestes polylepis* (0.1%–0.4%) is much less than intraspecific divergence (3.7%–4.3%) |
| NC_031633.1 *Thoburnia rhothoeca* | Deep intraspecific divergence (15.7%) |
| KJ631323.1; NC_030505.1 *Triplophysa orientalis* | Interspecific divergence with *Triplophysa stoliczkae* (5.5%) is much less than intraspecific divergence (17.6%) |
| KJ631324.1 *Triplophysa stewart* | Interspecific divergence with *Triplophysa stenura* (0.9%–3.5%) is much less than intraspecific divergence (9.3%) |
| KM396312.1 *Triplophysa robusta* | Deep intraspecific divergence (10.3%) |
| FJ406582.1–FJ406586.1; FJ406578.1–FJ406579.1; JQ686729.1 *Triplophysa bleekeri* | Deep intraspecific divergence (19.2%–20.5%) |
| KU870466.1 *Tor mosal mahanadicus* | Shallow interspecific divergence with *Tor putitora* (0.3%–1.8%) |
| KU314699.1 *Xenophysogobio boulengeri* | Deep intraspecific divergence (17.4%) |
| AY245022.1–AY245089.1 *Zacco platypus* | Deep intraspecific divergence (15.0%–17.4%) |
| KP738590.1 ; KP738589.1 *Acanthocobitis botia* | Deep intraspecific divergence (14.1%–14.3%) |
| KP015738.1 *Acheilognathus imberbis* | Deep intraspecific divergence (19.7%–20.2%) |
| AY116370.1 *Cottus poecilopus* | Deep intraspecific divergence (14.0%) |
| LC097819.1 –LC097824.1 *Cottus pollux* | Interspecific divergence with *Cottus reinii* (1.2%––1.9%) is much less than intraspecific divergence (13.3%–14.7%) |
| DQ683362.1 *Cromileptes altivelis* | Deep intraspecific divergence (10.9%) |
| AF099881.1–AF099884.1 *Crystallaria asprella* | Interspecific divergence with *Crystallaria cincotta* (0–0.3%) is much less than intraspecific divergence (12.8%–14.1%) |
| KF556648.1 *Epinephelus bleekeri* | Interspecific divergence with*Epinephelus fario* (0.8%–1%) is much less than intraspecific divergence (15.6%) |
| AY963559.1 *Epinephelus spilotoceps* | Shallow interspecific divergence with *Epinephelus fuscoguttatus* (0–2.1%) |
| JF742820.1; JF742819.1 *Etheostoma asprigene* | Interspecific divergence with *Etheostoma collettei* (5.4%) is much less than intraspecific divergence (7.4%––8.0%) |
| HQ128088.1 *Etheostoma bellator* | Interspecific divergence with *Etheostoma chermocki* (0.5%) is much less than intraspecific divergence (6.1–6.8%) |
| AY374261.1 *Etheostoma blennioides* | Interspecific divergence with *Etheostoma newmanii* (0.4%–0.9%) is much less than intraspecific divergence (8.3%–8.8%) |
| JX547432.1 *Etheostoma oophylax* | Interspecific divergence with *Etheostoma nigripinne* (0.1%–4.1%) is much less than intraspecific divergence (13.8%–14.5%) |
| HQ128114.1; AY374266.1 *Etheostoma coosae* | Deep intraspecific divergence (10.9%–12.2%) |
| JX547258.1 *Etheostoma crossopterum* | Interspecific divergence with *Etheostoma forbesi* (0–0.2%) is much less than intraspecific divergence (15.7%–16.3%) |
| AF411343.1–AF411358.1 *Etheostoma tetrazonum* | Interspecific divergence with *Etheostoma erythrozonum* (0–0.5%) is much less than intraspecific divergence (8.6%–9.3%) |
| HQ128131.1 *Etheostoma flabellare* | Deep intraspecific divergence (14.6%) |
| HQ128151.1; KF377177.1–KF377182.1 *Etheostoma kantuckeense* | Interspecific divergence with *Etheostoma tecumsehi* (1.7%–2.1%) is much less than intraspecific divergence (4.1%–4.8%) |
| GQ183672.1–GQ183677.1 *Etheostoma nigrum* | Interspecific divergence with *Etheostoma olmstedi* (0.1%–2.7%) is much less than intraspecific divergence (7.9%–8.4%) |
| GQ183694.1–GQ183700.1 *Etheostoma olmstedi* | Interspecific divergence with *Etheostoma perlongum* (2.3%–3.8%) is much less than intraspecific divergence (7.2%–8.2%) |
| FJ381056.1; FJ381042.1; FJ381043.1 *Etheostoma spectabile pulchellum* | Interspecific divergence with *Etheostoma whipplei* (0.4%–3.1%) is much less than intraspecific divergence (22.1%–23%) |
| HQ128180.1 *Etheostoma occidentale* | Shallow interspecific divergence with *Etheostoma simoterum atripinne* (0.5%–1.8) |
| HQ128184.1; HQ128183.1 *Etheostoma orientale* | Shallow interspecific divergence with *Etheostoma simoterum atripinne* (1.2%–2.2%) |
| FJ381007.1 *Etheostoma punctulatum* | Interspecific divergence with *Etheostoma mihileze* (0.2%–4.1%) is much less than intraspecific divergence (6.9%–7.2%) |
| HQ128208.1 *Etheostoma paludosum* | Shallow interspecific divergence with *Etheostoma radiosum* (0.1%–0.3%) |
| JF742805.1; JF742804.1 *Etheostoma virgatum* | Deep intraspecific divergence (11.6%–12.4%) |
| KC211159.1 *Percina uranidea* | Interspecific divergence with *Percina vigil* (0.3%–0.5%) is much less than intraspecific divergence (8.0%–8.3%) |
| HQ128276.1 *Percina tanasi* | Shallow interspecific divergence with *Percina uranidea* (0.2%–1.3%) |
| AY374287.1 *Percina ouachitae* | Shallow interspecific divergence with *Percina vigil* (0.1%–0.3%) |
| LC108091.1; LC108100.1; LC108052.1 *Pungitius kaibarae* | Interspecific divergence with *Pungitius sinensis* (1.1%–5%) is much less than intraspecific divergence (7.8% –10.1%) |
| LC108078.1; LC108077.1; LC108062.1; LC108061.1 *Pungitius sinensis* | Interspecific divergence with *Pungitius kaibarae* (0.4%–5.2%) is much less than intraspecific divergence (9.1%–9.6%) |
| KJ628001.1–KJ628009.1; KT989569.1 *Pungitius laevis* | Shallow interspecific divergence with *Pungitius pungitius* (0.7%–1.3%) |
| LC108108.1; LC108107.1; LC108104.1 *Pungitius tymensis* | Interspecific divergence with *Pungitius pungitius* (0.7%–1.5%) is much less than intraspecific divergence (7.4%–8.2%) |
| KC663435.1 *Sander canadensis* | Interspecific divergence with *Sander lucioperca* (0.1%–1.5%) is much less than intraspecific divergence (15.8%–16.3%) |
| LC108087.1; LC108050.1 *Pungitius pungiti*us | Interspecific divergence with *Pungitius tymensis* (0.7%–1.4%) is much less than intraspecific divergence (7.2%–7.5%) |
| EF439589.1; EF439588.1 *Serranus scriba* | Deep intraspecific divergence (16.3%) |
| AY786428.1 *Variola louti* | Interspecific divergence with *Variola albimarginata* (0.3%) is much less than intraspecific divergence (12%) |
| KJ561563.1 *Amphilius atesuensis* | Deep intraspecific divergence (19.1%–20.4%) |
| KJ561524.1 *Amphilius grammatophorus* | Deep intraspecific divergence (21%–22.5%) |
| KJ561523.1 *Amphilius rheophilus* | Interspecific divergence with *Amphilius grammatophorus* (0–4.2%) is much less than intraspecific divergence (22.2%–22.5%) |
| KJ561547.1; KJ561546.1 *Amphilius grandis* | Interspecific divergence with *Amphilius krefftii* (0–0.1%) is much less than intraspecific divergence (10.3%–10.5%) |
| KC331847.1 *Amphilius zairensis* | Interspecific divergence with *Amphilius laticaudatus* (2.2%) is much less than intraspecific divergence (11.3%) |
| KF862967.1–KF862969.1 *Arius maculatus* | Interspecific divergence with *Arius arius* (0.8%–1.1%) is much less than intraspecific divergence (7.4%–7.3%) |
| EU490904.1; AF416897.1 *Bagarius yarrelli* | Deep intraspecific divergence (10.4%–10.8%) |
| KF250576.1 *Chiloglanis brevibarbis* | Interspecific divergence with *Chiloglanis deckenii* (4.2%–4.3%) is much less than intraspecific divergence (8.5% –9.4%) |
| AB822528.1 *Clarias batrachus* | Deep intraspecific divergence (10.5%) |
| NC_004698.1 *Corydoras rabauti* | Interspecific divergence with *Corydoras zygatus* (0.5%–0.9%) is much less than intraspecific divergence (5.6%) |
| DQ192477.1; DQ192476.1 *Pareuchiloglanis kamengensis* | Interspecific divergence with *Creteuchiloglanis gongshanensis* (0.1%–2.5%) is much less than intraspecific divergence (5.9%–6.1%) |
| JQ026252.1 *Euchiloglanis kishinouyei* | Interspecific divergence with *Chiloglanis sinensis* (0.2%–0.9%) is much less than intraspecific divergence (7.2%) |
| DQ192461.1 *Exostoma labiatum* | Deep intraspecific divergence (12.2%–12.5%) |
| JN020081.1 ; JN020080.1 *Glyptothorax lampris* | Shallow interspecific divergence with *Glyptothorax laosensis* (0–0.1%) |
| JQ026262.1; JN986967.1 *Glyptothorax trilineatus* | Interspecific divergence with *Glyptothorax longicauda* (0.2%) is much less than intraspecific divergence (4.3%–4.4%) |
| FJ772196.1–FJ 772214.1 *Trichomycterus areolatus* | Interspecific divergence with *Hatcheria macraei* (0.8%–2.1%) is much less than intraspecific divergence (3.4%–4.2%) |
| AY912446.1 *Hemibagrus macropterus* | Interspecific divergence with *Hemibagrus guttatus* (0.2%–1.7%) is much less than intraspecific divergence (8.1%–8.3%) |
| AF499600.1 *Hemibagrus nemurus* | Interspecific divergence with *Hemibagrus wyckioides* (0.3%–0.5%) is much less than intraspecific divergence (15.8%) |
| NC_024273.1 *Hemibagrus nemurus* | Interspecific divergence with *Hemibagrus filamentus* (0.9%) is much less than intraspecific divergence (6.3%) |
| KJ624624.1 *Hemibagrus wyckioides* | Interspecific divergence with *Hemibagrus filamentus* (0.8%) is much less than intraspecific divergence (15.7%–16.2%) |
| AY327267.1 *Ictalurus lupus* | Interspecific divergence with *Ictalurus punctatus* (0.5%–1%) is much less than intraspecific divergence (8.3%) |
| AB045119.1 *Ictalurus punctatus* | Deep intraspecific divergence (23.7%–24.2%) |
| NC_021407.1 *Liobagrus nigricauda* | Shallow interspecific divergence with *Liobagrus marginatoides* (0–0.1%) |
| JN986968.1 *Liobagrus nigricauda* | Shallow interspecific divergence with *Liobagrus anguillicauda* (0–0.1%) |
| DQ192462.1; KC757128.1 *Liobagrus marginatus* | Interspecific divergence with *Liobagrus marginatoides* (0–0.1%) is much less than intraspecific divergence (10.6%–11.2%) |
| KX265430.1 *Liobagrus somjinensis* | Shallow interspecific divergence with *Liobagrus mediadiposal* (0.5%–0.9%) |
| LC064950.1––LC064961.1 *Liobagrus reinii* | Deep intraspecific divergence (9.5%–10.1%) |
| KF862960.1 *Mystus bleekeri* | Deep intraspecific divergence (16.5%–17.4%) |
| KU870465.1 *Mystus cavasius* | Deep intraspecific divergence (10.9%–11%) |
| HQ257293.1 *Mystus gulio* | Deep intraspecific divergence (20.8–21.4%) |
| JQ343984.1 *Mystus rhegma* | Interspecific divergence with *Mystus mysticetus* (0.3%–0.5%) is much less than intraspecific divergence (16.6%) |
| KF862948.1 *Neotropius atherinoides* | Interspecific divergence with *Mystus dibrugarensis* (5.3%–5.6%) is much less than intraspecific divergence (8.0%) |
| AY327289.1 *Noturus flavus* | Deep intraspecific divergence (9.8%–10.0%) |
| FJ013166.1 *Netuma thalassina* | Deep intraspecific divergence (11.8%) |
| AY458897.1 *Pangasius sutchi* | Shallow interspecific divergence with *Pangasianodon hypophthalmus* (0.1%) |
| JN020087.1; JN020086.1 *Pangasius sanitwongsei* | 100% identify with *Pangasianodon hypophthalmus* but deep intraspecific divergence (13.9%–14.2%) |
| DQ192477.1; DQ192476.1 *Pareuchiloglanis kamengensis* | Interspecific divergence with *Pareuchiloglanis longicauda* (1.7%–1.8%) is much less than intraspecific divergence (5.9%–6.2%) |
| KJ637323.1 *Pareuchiloglanis sinensis* | Interspecific divergence with *Pareuchiloglanis longicauda* (0.1%) is much less than intraspecific divergence (12.8%–13.6%) |
| KJ001784.1 *Pelteobagrus eupogon* | Deep intraspecific divergence (13.9%–15%) |
| \| AY912322.1 *Pelteobagrus fulvidraco* \| \| --- \| | Interspecific divergence with *Pelteo bagrus nitidus* (5.1%–5.5%) is much less than intraspecific divergence (7.9%––8.3%) |
| AJ245673.1; AJ245638.1 *Schilbe intermedius* | 100% identify with *Schilbe mystus* but deep intraspecific divergence (3.5%) |
| LC098650.1; LC098649.1; LC098642.1; JX256247.1 *Silurus asotus* | Interspecific divergence with *Silurus lithophilus* (0.7%–1.2%) is much less than intraspecific divergence (5.8% ––6.3%) |
| KP255959.1; HQ890503.1 *Silurus lanzhouensis* | Deep intraspecific divergence (12.1%–12.5%) |
| AB860299.1 *Silurus soldatovi* | Shallow interspecific divergence with *Silurus asotus* (0.4%–1.4%) |
| HF566072.1 *Synodontis schoutedeni* | Interspecific divergence with *Synodontis smiti* (0.3%) is much less than intraspecific divergence (10.3%) |
| AY912439.1; AY912438.1 *Leiocassis crassilabris* | Interspecific divergence with *Pseudobagrus emarginatus* (0.4%–0.5%) is much less than intraspecific divergence (9.0%–9.8%) |
| AF499597.1; AY912381.1–AY912384.1 *Pseudobagrus tenuis* | Interspecific divergence with *Pseudobagrus ussuriensis* (0–2.2%) is much less than intraspecific divergence (7.7%) |
| AF416895.1 *Pseudobagrus truncatus* | Interspecific divergence with *Leiocassis crassilabris* (0.5%–0.7%) is much less than intraspecific divergence (10.1% –10.3%) |
| JX867258.1 *Pseudobagrus ussuriensis* | Interspecific divergence with *Pseudobagrus truncatus* (0.1%–1.4%) is much less than intraspecific divergence (9.9%–10.4%) |
| AF416880.1 *Pseudobagrus brevicaudatus* | Interspecific divergence with *Pseudobagrus truncatus* (0.1%–1.4%) is much less than intraspecific divergence (9.7%) |
| AF510817.1 *Chapalichthys encaustus* | Interspecific divergence with *Skiffia bilineata* (0.1%) is much less than intraspecific divergence (6.9%–7.1%) |
| GQ119684.1; GQ119683.1 *Fundulus bermudae* | Interspecific divergence with *Fundulus heteroclitus* (0.5%–2.1%) is much less than intraspecific divergence (5.1%–5.4%) |
| GQ119688.1 *Fundulus blairae* | Interspecific divergence with *Fundulus dispar* (0.5%) is much less than intraspecific divergence (5.8%–6.4%) |
| GQ119707.1 *Fundulus dispar* | Interspecific divergence with *Fundulus blairae* (1.8%–2.0%) is much less than intraspecific divergence (5%) |
| KJ696801.1 *Fundulus cingulatus* | Interspecific divergence with *Fundulus chrysotus* (0.9%–2.0%) is much less than intraspecific divergence (19.4%) |
| KP059009.1 *Fundulus notatus* | Interspecific divergence with *Fundulus escambiae* (1.0%–2.0%) is much less than intraspecific divergence (18.2%–18.7%) |
| KX359047.1; KX359046.1 *Fundulus zebrinus* | Interspecific divergence with *Fundulus kansae* (0–2.6%) is much less than intraspecific divergence (9.5%–10.3%) |
| KP059011.1 *Gambusia affinis* | Shallow interspecific divergence with *Fundulus olivaceus* (0.1%–1.9%) |
| KJ696832.1 *Poecilia petenensis* | Interspecific divergence with *Poecilia mexicana* (1.7%–2.0%) is much less than intraspecific divergence (9.2%–9.3%) |
| KP699889.1 *Poecilia latipinna* | Interspecific divergence with *Poecilia petenensis* (0.1%–0.2%) is much less than intraspecific divergence (6.2%–6.5%) |
| J696840.1 *Poecilia sphenops* | Interspecific divergence with *Poecilia mexicana* (0.7%––2%) is much less than intraspecific divergence (8.7%–9.2%) |
| NC_026579.1 *Poecilia sphenops* | Interspecific divergence with *Poecilia latipinna* (0–0.9%) is much less than intraspecific divergence (8.7%–9.4%) |
| JQ012427.1 *Engraulis eurystole* | Shallow interspecific divergence with *Engraulis encrasicolus* (0.9%–3.0%) |
| AY912355.1 –AY912357.1 *Pelteobagrus nitidus* | Interspecific divergence with *Pseudobagrus ussuriensis* (0.1%–0.8%) is much less than intraspecific divergence (8.9%–9.4%) |
| AY912380.1; AY912379.1 *Pseudobagrus tenuis* | Interspecific divergence with *Pelteobagrus nitidus* (0.3%–1.1%) is much less than intraspecific divergence (7.8%–8.3%) |
| HF566007.1 *Synodontis caudovittata* | Shallow interspecific divergence *with Synodontis frontosa* (0.4%–1.0%) |
| KC778794.1 *Skiffia bilineata* | Interspecific divergence wit*h Neotoca bilineata* (0–0.7%) is much less than intraspecific divergence (12.1%) |
| JQ612851.1 *Pseudoxiphophorus bimaculatu* | Interspecific divergence with *Pseudoxiphophorus jonesii* (1.4%–4.4%) is much less than intraspecific divergence (8.2%–9.2%) |
| JQ612853.1 –JQ612855.1 *Pseudoxiphophorus jonesii* | Deep intraspecific divergence (9.2%–10.6%) |
| KC778798.1 *Xenotoca variata* | Shallow interspecific divergence with *Xenotoca variatus* (0.9%–1.5%) |
| KJ696849.1 *Xiphophorus alvarezi* | Shallow interspecific divergence with *Xiphophorus hellerii* (0.1%–0.5% ) |
| AY164465.1 *Dicologlossa cuneata* | Interspecific divergence with divergence *Microchirus azevia* (0.2%–0.7%) is much less than intraspecific divergence (24.5%–24.8%) |
| EU224027.1 *Platichthys flesus* | Deep intraspecific divergence (26.5% –26.7%) |
| DQ464116.1 *Pseudopleuronectes obscurus* | Interspecific divergence with *Pseudopleuronectes yokohamae* (0.3%–3.5%) is much less than intraspecific divergence (5.6%–5.7%) |
| EF392604.1; AB125325.1; EF392603.1 *Solea lascaris* | Deep intraspecific divergence (16.2%–16.7%) |
| DQ198003.1 *Solea solea* | Deep intraspecific divergence (23.2%–24%) |
| AY164468.1 *Synaptura lusitanica* | Interspecific divergence with *Psetta maxima* (0.1%–0.9%) is much less than intraspecific divergence (37%) |
| JQ012422.1 –JQ012423.1 *Anchoviella carrikeri* | Deep intraspecific divergence (11.9%–12.1%) |
| EU694408.1; EU694407.*1 Coilia mystus* | Interspecific divergence with *Coilia nasus* (0–0.4%) is much less than intraspecific divergence (8.7%–9.2%) |
| JX030395.1–JX030396.1; KF056322.1 *Coilia mystus* | Deep intraspecific divergence (10.1%–10.6%) |
| NC_009583.1 *Etrumeus teres* | Deep intraspecific divergence (15.7%) |
| NC_016698.1 *Potamothrissa obtusirostris* | Deep intraspecific divergence (20.9%) |
| AF472586.1 Sardinops sagax | Interspecific divergence with *Sardinops caeruleus* (0.7%) is much less than intraspecific divergence (2.8%–3.0%) |
| JQ012365.1 *Setipinna taty* | Deep intraspecific divergence (17.6%–17.7%) |
| KU761588.1 *Thryssa kammalensis* | Interspecific divergence with *Coilia nasus* (1.5%–2%) is much less than intraspecific divergence (19.9%) |
| FN813017.1 *Mastacembelus brachyrhinus* | Deep intraspecific divergence (15.5%–15.7%) |
| KT732480.1 KT732479.1 *Mastacembelus niger* | Deep intraspecific divergence (10.1%–11.7%) |
| AY355060.1–AY355073.1 *Ophisternon aenigmaticum* | Deep intraspecific divergence (13.9–16.2%) |
| AY355128.1 –AY355137.1 *Synbranchus marmoratus* | Deep intraspecific divergence (12.0%–17.0%) |
| EF392567.1 *Aluterus scriptus* | Deep intraspecific divergence (23.5%) |
| KF025770.1 *Cantherhines pullus* | Shallow interspecific divergence with *Cantherhines pardalis* (0–0.4%) |
| AM265578.1 *Mola mola* | Interspecific divergence with *Masturus lanceolatus* (0.1%) is much less than intraspecific divergence (13.1%–13.4% ) |
| KF027567.1 JQ681927.1 *Monotrete leiurus* | Interspecific divergence with *Monotrete cochinchinensis* (0–2.3%) is much less than intraspecific divergence (4.3%) |
| JQ681932.1 *Tetraodon palembangensis* | Interspecific divergence with *Monotrete cochinchinensis* (0–2.5%) is much less than intraspecific divergence (13%) |
| JQ681908.1 *Sphoeroides parvus* | Interspecific divergence with *Sphoeroides dorsalis* (0.3%) is much less than intraspecific divergence (14.5%) |
| NC_004416.1 *Sufflamen fraenatum* | Interspecific divergence with *Sufflamen verres* (3.1%) is much less than intraspecific divergence (7.1%) |
| FJ434545.1 *Takifugu stictonotus* | Interspecific divergence with *Takifugu snyderi* (0.1%) is much less than intraspecific divergence (2.3%) |
| JQ681940.1 *Tylerius spinosissimus* | Deep intraspecific divergence (12.1%) |
| JQ312524.1; JQ312515.1; JQ312487.1; JQ312433.1; JQ312442.1; JQ312444.1; JQ312448.1; JQ312451.1; JQ312452.1; JQ312455.1; JQ312456.1; JQ312459.1; JQ312460.1; JQ312462.1 *Agonostomus monticola* | Deep intraspecific divergence (14.8%–15.4%) |
| EU036450.1; EU036449.1 *Mugil cephalus* | Deep intraspecific divergence (10.5%–11.1%) |
| KJ713912.1; KJ713899.1 *Campylomormyrus alces* | Interspecific divergence with *Campylomormyrus elephas* (0.7% –1%) is much less than intraspecific divergence (3.0%–3.1%) |
| KJ713930.1 *Campylomormyrus alces* | Interspecific divergence with *Campylomormyrus tamandua* (0.2%–1.4%) is much less than intraspecific divergence (5.4% –5.5%) |
| AB035245.1 *Campylomormyrus elephas* | Interspecific divergence with *Campylomormyrus compressirostris* (0.2%–0.5%) is much less than intraspecific divergence (3.5%–4.2% ) |
| AJ245679.1 *Marcusenius macrolepidotus* | Interspecific divergence with *Marcusenius macrolepidotis* (0–2.1%) is much less than intraspecific divergence (4.3%) |
| GU997128.1 *Pantodon buchholzi* | Deep intraspecific divergence (17.8%–18.1%) |
| KM361633.1*Coregonus peled* | Interspecific divergence with *Coregonus lavaretus* (0.2%–1.3%) is much less than intraspecific divergence (3.9%–4.3%) |
| KU530206.1 *Coregonus chadary* | Interspecific divergence with *Coregonus nasus* (0–0.8%) is much less than intraspecific divergence (2.6%) |
| JX960774.1; JX960773.1; AJ617503.1; AJ251591.1 *Coregonus autumnalis* | Interspecific divergence with *Coregonus artedi* (0.7%–1.4%) is much less than intraspecific divergence (3.9%–4.6%) |
| EU492281.1 *Salmo salar* | Interspecific divergence with *Salmo trutta* (0–0.3%) is much less than intraspecific divergence (5.7%–6.4%) |
| JX960862.1 *Thymallus arcticuswith* | Interspecific divergence with *Thymallus baicalolenensis* (0.1%) is much less than intraspecific divergence (3.6%–3.7%) |
| JX960867.1; KF649073.1 *Thymallus grubii* | Interspecific divergence with *Thymallus tugarinae* (0.8%–1%) is much less than intraspecific divergence (7.6%–7.9%) |
| HQ325615.1 *Cheilopogon furcatus* | Interspecific divergence with *Cheilopogon melanurus* (0.1%–0.3%) is much less than intraspecific divergence (4.3%) |
| NC_003184.1 *Exocoetus volitans* | Interspecific divergence with *Exocoetus peruvianus* (0.1%–0.9%) is much less than intraspecific divergence (8.7%–9%) |
| LC051677.1; LC051676.1*Oryzias wolasi* | Interspecific divergence with *Oryzias asinua* (0.7%) is much less than intraspecific divergence (3.4%–3.6%) |
| KP200665.1–KP200668 *Salvelinus malma* | Interspecific divergence with *Salvelinus curilus* (0.1%–0.3%) is much less than intraspecific divergence (2.4%–2.7%) |
| AB084751.1; AB100938.1–AB100951.1; AB455001.1–AB455003.1 *Oryzias latipes* | Deep intraspecific divergence (15.7%–17.4%) |
| HQ325660.1 *Parexocoetus mento* | Interspecific divergence with *Parexocoetus brachypterus* (1.0%–1.2%) is much less than intraspecific divergence (13.2%) |
| KJ564271.1 *Anguilla rostrata* | Interspecific divergence with *Anguilla anguilla* (0–1.7%) is much less than intraspecific divergence (4.5%–4.9% ) |
| KJ948424.1 *Anguilla japonica* | Deep intraspecific divergence (11.6%–11.9%) |
| AF006719.1; AF006718.1 *Anguilla malgumora* | Interspecific divergence with *Anguilla luzonensis* (0.3%–1%) is much less than intraspecific divergence (8.8%–9%) |
| DQ197953.1 *Gymnothorax maderensis* | Deep intraspecific divergence (14.4%––14.7%) |
| DQ536423.1 *Lepisosteus osseus* | Interspecific divergence with *Lepisosteus oculatus* (0–5.4%) is much less than intraspecific divergence (10.6%–10.9%) |
| EU253550.1; EU253549.1 *Atherina boyeri* | Deep intraspecific divergence (10.2%–10.8%) |
| GU932753.1 *Atherinomorus lacunosus* | Interspecific divergence with *Atherinomorus forskalii* (0–0.5%) is much less than intraspecific divergence (13.3%) |
| GU932791.1; GU932788.1; GU932787.1 *Craterocephalus stramineus* | Deep intraspecific divergence (10.7%–13.0%) |
| KC133605.1 *Glossolepis incisus* | Interspecific divergence with *Glossolepis dorityi* (0.1%) is much less than intraspecific divergence (4.7%) |
| KP345856.1 *Melanotaenia albimarginata* | Interspecific divergence with *Melanotaenia aruensis* (0.2%) is much less than intraspecific divergence (8.7%) |
| KC133563.1 *Melanotaenia australis* | Interspecific divergence with *Melanotaenia exquisita* (1.3%) is much less than intraspecific divergence (10.6%–10.9%) |
| KU529305.1 *Melanotaenia australis* | Interspecific divergence with *Melanotaenia nigrans* (1.7%–2.2%) is much less than intraspecific divergence (11.9%–11.8%) |
| KC133618.1 *Melanotaenia boesemani* | Interspecific divergence with *Melanotaenia ajamaruensis* (0.3%–0.4%) is much less than intraspecific divergence (14.7%) |
| KC133576.1 *Melanotaenia lacustris* | Interspecific divergence with *Melanotaenia boesemani* (0%) is much less than intraspecific divergence (4.9%) |
| KC133538.1 *Melanotaenia trifasciata* | Deep intraspecific divergence (10%–10.4%) |
| NC_004385.1 *Melanotaenia lacustris* | Shallow interspecific divergence with *Melanotaenia trifasciata* (0.7%–1.3%) |
| KC133624.1 *Melanotaenia misoolensis* | Interspecific divergence with *Melanotaenia flavipinnis*  (0.2%–0.3%) is much less than intraspecific divergence (3.1%) |
| KU833247.1–KU833250 *Pseudomugil gertrudae* | Interspecific divergence with *Pseudomugil paskai* (0.5%–2.8%) is much less than intraspecific divergence (8.4%–9.2%) |
| AH012866.2 *Brosme brosme* | Deep intraspecific divergence (12.4%–12.7%) |
| AB366447.1; KM408078.1 *Acanthorhodeus chankaensis* | Interspecific divergence with *Acheilognathus gracilis* (0.7%–0.8) is much less than intraspecific divergence (4.7%–5.1%) |
| AB366538.1 *Acheilognathus somjinensis* | Interspecific divergence with *Tanakia koreensis* (0.5%–1.5%) is much less than intraspecific divergence (10.9%–11.2%) |
| KF410717.1; KF410716.1 *Acheilognathus gracilis* | Interspecific divergence with *Acanthorhodeus chankaensis* (0.7%–6.8%) is much less than intraspecific divergence (16.9%–18.0%) |
| GU932753.1 *Atherinomorus lacunosus* | Interspecific divergence with *Atherinomorus forskalii* (0%–0.5%) is much less than intraspecific divergence (13.3%) |
| AF145923.1 *Barbus setivimensis* | Interspecific divergence with *Barbus magniatlantis* (3.4%–3.8%) is much less than intraspecific divergence (9.4%) |
| EU082477.1 *Campostoma pullum* | Interspecific divergence with *Campostoma plumbeum* (0.2%) is much less than intraspecific divergence (9.0%) |
| AF454872.1 *Catostomus microps* | Interspecific divergence with *Catostomus tahoensis* (0.9%–1.1%) is much less than intraspecific divergence (11.3%) |
| KP115291.1 *Devario laoensis* | Interspecific divergence with *Devario chrysotaeniatus* (7.8%) is much less than intraspecific divergence (14.7%) |
| HQ128075.1 *Etheostoma artesiae* | Interspecific divergence with *Etheostoma swaini* (5.4%–7.6%) is much less than intraspecific divergence (10.3%–10.4%) |
| JX547246.1; JX547256.1; JX547253.1 *Etheostoma crossopterum* | Interspecific divergence with *Etheostoma nigripinne* (0%–0.3%) is much less than intraspecific divergence (15.6%–16.8%) |
| KF377102.1; KF377105.1; KF377100.1; KF377099.1; KF377098.1; KF377097.1 *Etheostoma spectabile spectabile* | Interspecific divergence with *Etheostoma caeruleum* (0.1%–2.8%) is much less than intraspecific divergence (22.3%–22.8%) |
| JX547422.1 *Etheostoma squamiceps* | Interspecific divergence with *Etheostoma oophylax* (0.3%–1.5%) is much less than intraspecific divergence (16.6%–17.4%) |
| KT833112.1*Gymnodiptychu spachycheilus* | Interspecific divergence with *Schizopygopsis stoliczkai* (4.7%–5.1%) is much less than intraspecific divergence (15.7%–15.9%) |
| DQ026432.1 *Gyrinocheilus aymonieri* | Interspecific divergence with *Gyrinocheilus pennocki* (0.1%) is much less than intraspecific divergence (11.2%) |
| HQ235749.1 *Garra mirofrontis* | Interspecific divergence with *Pseudogyrinocheilus prochilus* (3.5%–3.6%) is much less than intraspecific divergence (13.0%–13.1%) |
| GU932757.1 *Hypoatherina temminckii* | Interspecific divergence with *Hypoatherina golanii* (0.2%) is much less than intraspecific divergence (15.6%–17%) |
| KT833108.1; KT833082.1 *Herzensteinia microcephalus* | Interspecific divergence with *Gymnocypris namensis* (0.4%–0.7%) is much less than intraspecific divergence (4.7%–5.5%) |
| AJ251092.1 *Leuciscus zrmanjae* | Interspecific divergence with *Leuciscus cephalus* (0.4%–0.5%) is much less than intraspecific divergence (8.7%) |
| KC133541.1 *Melanotaeniaru brostriatus* | Interspecific divergence with *Melanotaenia splendida* (2.1%–2.7%) is much less than intraspecific divergence (7.2%–8.8%) |
| KP644326.1 *Micromesistius poutassou* | Interspecific divergence with *Melanogrammus aeglefinus* (6.1%–6.2%) is much less than intraspecific divergence (18%–18.9%) |
| NC_029442.1 *Osteochilus hasseltii* | Interspecific divergence with *Osteochilus vittatus* (0%) is much less than intraspecific divergence (10%) |
| JN003327.1; AY953004.1 *Romanogobio tenuicorpus* | Interspecificdivergence with *Gobio cynocephalus* (8.2%–8.3%) is much less than intraspecific divergence (19.4%–19.5%) |
| EU241464.1 *Rasbora brittani* | Interspecific divergence with *Pectenocypris korthausae* (0%–1.6%) is much less than intraspecific divergence (21.5%) |
| JQ651590.1; JQ651586.1; JQ651584.1; JQ651571.1; JQ651569.1 *Telestes muticellus* | Interspecific divergence with *Telestes souffia* (0%–0.1%) is much less than intraspecific divergence (9.8%–10.5%) |
| FJ694975.1 *Zacco pachycephalus* | Deep intraspecific divergence (14.9%–15.8%) |
